# Gastrointestinal and hepatic manifestations of COVID‐19: A systematic review and meta‐analysis

**DOI:** 10.1002/jgh3.12456

**Published:** 2020-11-21

**Authors:** Robert D Dorrell, Michael K Dougherty, Eric L Barash, Asher E Lichtig, Steven B Clayton, Elizabeth T Jensen

**Affiliations:** ^1^ Department of Medicine Medical Center Blvd Winston‐Salem North Carolina USA; ^2^ Division of Gastroenterology and Hepatology University of North Carolina at Chapel Hill Chapel Hill North Carolina USA; ^3^ Department of Wake Forest School of Medicine Medical Center Blvd Winston‐Salem North Carolina USA; ^4^ Department of Epidemiology and Prevention Medical Center Blvd Winston‐Salem North Carolina USA; ^5^ Department of Medicine, Section on Gastroenterology Medical Center Blvd Winston‐Salem North Carolina USA

**Keywords:** COVID‐19, gastrointestinal, meta‐analysis, SARS‐CoV‐2, systematic review

## Abstract

**Background and Aim:**

This review investigates the role of gastrointestinal and hepatic manifestations in COVID‐19, particularly with regard to the prevalence of isolated gastrointestinal (GI) symptoms.

**Methods:**

We searched PubMed, Embase, and Cochrane library for COVID‐19 publications from 1 December 2019 to 18 May 2020. We included any study that reported the presence of GI symptoms in a sample of >5 COVID‐19 patients. Data collection and risk of bias assessment were performed independently by two reviewers. Where ≥3 studies reported data sufficiently similar to allow calculation of a pooled prevalence, we performed random effects meta‐analysis.

**Results:**

This review included 17 776 COVID‐19 patients from 108 studies. Isolated GI symptoms only occurred in 1% (95% confidence interval [CI] 0–6%) of patients. GI symptoms were reported in 20% (95% CI 15–24%) of patients. The most common were anorexia (21%, 95% CI 15–27%), diarrhea (13%, 95% CI 11–16%), nausea or vomiting (8%, 95% CI 6–11%), and abdominal pain (4%, 95% CI 2–6%). Transaminase elevations were present in 24% (95% CI 17–31%) of patients. Higher prevalence of GI symptoms were reported in studies published after 1st April, with prevalence of diarrhea 16% (95% CI 13–20), nausea or vomiting 12% (95% CI 8–16%), and any GI symptoms 24% (95% CI 18–34%). GI symptoms were associated with severe COVID‐19 disease (odds ratio [OR] 2.1, 95% CI 1.3–3.2), but not mortality (OR 0.90, 95% CI 0.52–1.54).

**Conclusions:**

Patients with isolated GI symptoms may represent a small but significant portion of COVID‐19 cases. When testing resources are abundant, clinicians should still consider testing patients with isolated GI symptoms or unexplained transaminase elevations for COVID‐19. More recent studies estimate higher overall GI involvement in COVID‐19 than was previously recognized.

## Graphical abstract

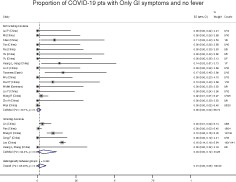

This review is a systematic review and meta‐analysis to characterize the prevalence of gastrointestinal (GI) and hepatic involvement in COVID‐19 and specifically assess the prevalence of GI symptoms in isolation. This review included 17 776 COVID‐19 patients from 108 studies. Isolated GI symptoms occurred in 1% (95% confidence interval 0–6%) of patients.

## Introduction

The novel coronavirus SARS‐CoV‐2, also known as coronavirus 2019 (COVID‐19), originated in Wuhan, China during the fall of 2019 before rapidly disseminating throughout the world and being declared a pandemic by the World Health Organization in March, 2020. COVID‐19 was initially considered a primary respiratory illness with symptoms of shortness of breath, cough, and fever. However, there is an increasing appreciation of the ability of SARS‐CoV‐2 to produce a wide range of symptoms,^1^ including GI (gastrointestinal) symptoms like diarrhea, nausea, vomiting, and abdominal pain.^2^

Current CDC testing guidelines for COVID‐19 recognize the diversity of symptoms possible and advise testing for patients displaying fever, cough, shortness of breath, chills, myalgia, new loss of taste or smell, vomiting, diarrhea, or sore throat.^1^ In practice, respiratory symptoms are prioritized by both patients and healthcare workers for testing and pre‐emptive isolation precautions.^3^ In accordance with the worldwide effort to arrest the spread of this virus and mitigate its effects on the global population, the aim of this systematic review and meta‐analysis is to determine^1^ whether isolated GI symptoms warrant testing for COVID‐19, and^2^ whether GI symptoms, alone or in combination with respiratory symptoms, are associated with severe disease and/or mortality.

## Methods

### Search strategy and study selection

PubMed, Embase, and the Cochrane library were searched from 1 December 2019 to 18 May 2020 with the help of a medical librarian (full search terms and inclusion criteria in supplement). The study protocol was registered with PROSPERO (CRD42020182644).

We included any study that reported the presence or absence of GI manifestations in a sample of ≥5 COVID‐19 positive patients, including diarrhea, nausea, vomiting, anorexia (loss of appetite), abdominal pain, or liver injury, with or without the presence of typical respiratory viral symptoms. Two authors independently reviewed each article for inclusion, extracted data, and assessed the risk of bias (ROB) using the Newcastle Ottawa Scale.

### Quantitative analysis

Where ≥3 studies reported data sufficiently similar to allow calculation of a pooled prevalence, we performed random effects meta‐analysis. The primary outcome was the proportion of COVID‐19 positive patients who experienced a period of isolated GI symptoms. We also calculated pooled proportions of patients with any experience of a GI symptom including diarrhea, abdominal pain, nausea/vomiting, and anorexia (loss of appetite). We performed a separate analysis of proportions of COVID‐19 positive patients with elevated alanine transaminase (ALT) or aspartate transaminase (AST) as well as pooled mean AST and ALT, weighted according to number of patients in each study with transaminase measurements. In a secondary analysis, we calculated the odds ratio (OR) of severe COVID‐19 *versus* non‐severe COVID‐19 based on the presence of GI symptoms. The Freeman‐Tukey double arcsine transformation was used in the meta‐analysis of proportions.^4^ Heterogeneity was assessed qualitatively and quantitatively (using the I^2^ statistic) and explored using stratified and subgroup analyses. Covariates included risk of bias, study location, publication date, type of healthcare setting (inpatient *vs* outpatient), COVID‐19 case definition, pediatric *versus* adult patients, and whether the study focused on a unique subpopulation or health condition. We assessed for small‐study effects in the primary analysis visually with funnel plots, as well as the Egger test for funnel plot asymmetry. Analyses were performed in STATA 14.2 (College Station, TX, USA). Additional details of study selection and analysis are described in eMethods.

## Results

### Search results and study characteristics

**Figure 1:**
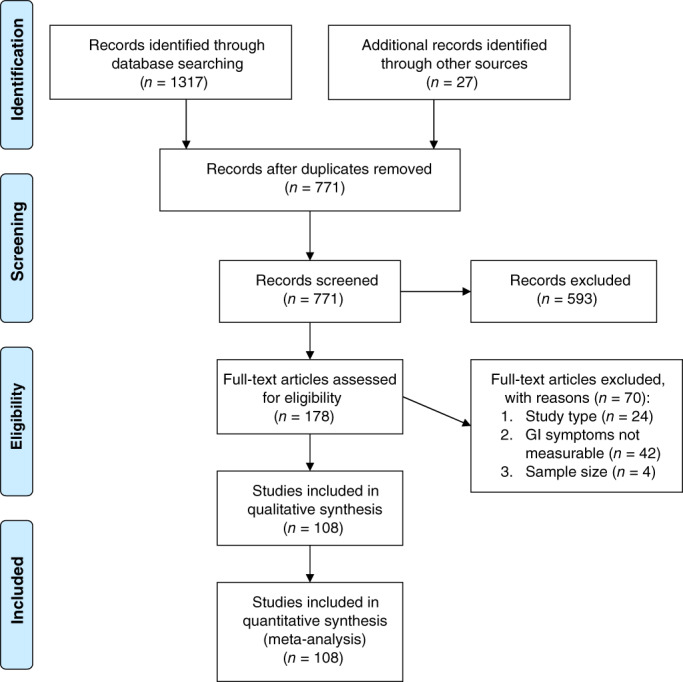
PRISMA flow diagram.

Our search identified 771 unique articles, of which 108 met inclusion criteria (Fig. 1). These comprised 17 776 COVID‐19 positive patients from 16 countries on three continents, including 13 769 patients from Asia. (Table S1, Supporting information) The majority of studies focused on adult patients (78 studies) and the hospital setting (102 studies). Many studies focused on specific hospitalized subpopulations, such as healthcare workers, family clusters, and orthopedic, pregnant, or critically ill patients (Table S1). All but nine studies^5^, ^6^, ^7^, ^8^, ^9^, ^10^, ^11^, ^12^, ^13^ defined COVID‐19 cases exclusively by positivity on an upper respiratory swab polymerase chain reaction (PCR). There were only three prospective studies,^14^, ^15^, ^16^ and none were determined to have low risk of bias (Table S2).

### *Prevalence of luminal* *GI* *symptoms*

The most common GI symptoms recorded in studies were diarrhea (98 studies), nausea and/or vomiting (69 studies), abdominal pain (39 studies), and anorexia/loss of appetite (22 studies). The overall prevalence of any GI symptom in COVID‐19 patients was 20% (95% CI 15–24%) using the definitions of each study, though with a very large number of studies and consequent heterogeneity (I^2^ 94%). When “loss of appetite” was not included in the definition of GI symptoms (leaving only diarrhea, abdominal pain, and nausea or vomiting), GI symptoms only occurred in 17% (95% CI 13–21%), *vs* 32% (95% CI 22–43%) when including anorexia as a GI symptom (*P* = 0.01, Figure S1). Findings were similar when examining experience of GI symptoms specifically as part of initial presentation (19%, 95% CI 14–25%, Figure S2).

**Figure 2:**
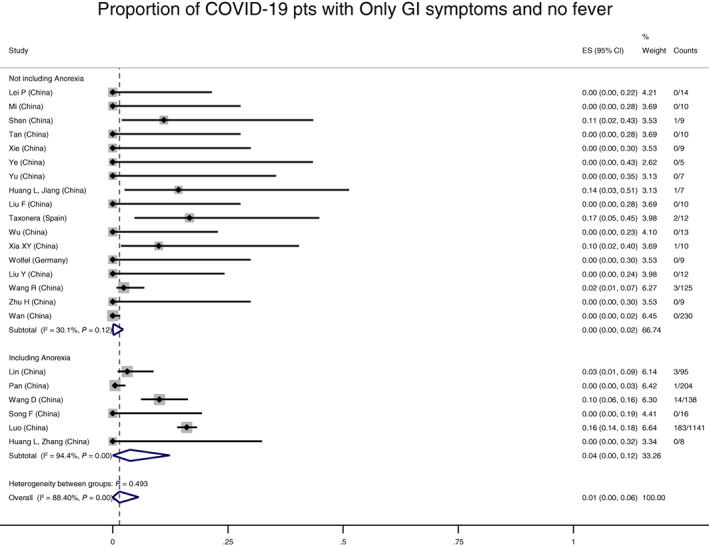
Proportion of coronavirus disease (COVID)‐19 patients with only gastrointestinal (GI) symptoms, without fever. Overall prevalence of COVID‐19 infection with a period of only GI symptoms without fever was 1% (95% confidence interval 0–6%). Whether anorexia was included in the definition of GI symptoms did not affect the proportion with GI symptoms in a statistically significant manner (*P* = 0.49).

Twenty‐five studies, including 2870 COVID patients, reported on our primary outcome of patients who endorsed isolated GI symptoms at any point in the disease course without concurrent respiratory symptoms. Overall 2% (95% CI 0–6%, I^2^ 63%) of COVID‐19 patients experienced isolated GI symptoms, with negligible difference whether the definition included anorexia (Figure S3) or whether the allowance of fever was defined (Figure S4). One percent (95% CI 0–6%, I^2^ 88%) had only GI symptoms without fever, with greater heterogeneity and a non‐significant trend toward higher prevalence if anorexia was included (0%, 95% CI 0–2%, I^2^ 30% if no anorexia; 4%, 95% CI 0–12%, I^2^ 94% if anorexia included, Fig. 2). The prevalence of GI symptoms in 124 studies that reported GI symptoms as the only symptom at first illness presentation was also similar (4%, 95% CI 0–10%, I^2^ 94%). GI symptoms lasted a mean of 4.6 (±1.9) days, based on four studies, but patients presented later in their disease course (mean of 13.2 [±5.4] days after onset of first symptoms). No study reported on a cohort of patients who were definitively known to never have developed respiratory or systemic symptoms. Notably, several^17^ studies required the presence of respiratory symptoms for inclusion, and therefore would not have detected isolated GI symptoms. Likewise, there were also studies focused on GI symptoms that did not report an overall denominator of COVID‐19 positive patients.^3^, ^18^, ^19^, ^20^, ^21^, ^22^ In analyses of funnel plots, there was no evidence of small‐study effects (such as publication bias) for the outcome of proportion of patients with only GI symptoms (Egger test *P* = 0.12, Figure S5).

Regarding specific luminal symptoms, diarrhea occurred in 13% (95% CI 11–16%, I^2^ 94%) of COVID‐19 positive individuals, with significantly lower rates from studies examining cohorts identified by respiratory symptoms (9% *vs* 15% in cohorts not restricted to respiratory syndromes, *P* < 0.01, Figure S6). Nausea or vomiting was present in 10% (95% CI 7–12%, I^2^ 94%, Figure S7) of patients. We assumed that all who vomited also had nausea, but the proportion of patients from 34 studies specifically reporting vomiting was 4% (95% CI 3–6%, I^2^ 83%, Figure S8). Abdominal pain occurred in 4% (95% CI 2–6%, I^2^ 93%, Figure S9) of patients, and anorexia in 21% (95% CI 15–27%, I^2^ 97%, Figure S10). Diarrhea, when defined, was usually ≥3 loose bowel movements per day, but few studies quantified the severity of any GI symptoms.

In subgroup analyses to explore heterogeneity, the most significant covariates were geography and date of publication. Studies from Europe or North America reported higher proportions of all GI symptoms, which in bivariate analyses were statistically significant for diarrhea (Asia 11%, 95% CI 9–14%; West 24%, 95% CI 18–31%, Figure S11), nausea or vomiting (Asia 8%, 95% CI 6–10%; West 18%, 95% CI 15–22%, Figure 12), abdominal pain (Asia 2%, 95% CI 1–3%; West 12%, 6–20%, Figure S13), and any GI symptoms (Asia 17%, 95% CI 13–22%; West 31%, 95% CI 20–43%, Figure S14), though not for anorexia (*P* = 0.14, Figure S15). These results were driven by the majority of Asian studies from China, with similarly significant differences when comparing Chinese *versus* non‐Chinese studies (including 5 studies total from Singapore, Korea, Taiwan, and Macau).^4^, ^23^, ^24^, ^25^, ^26^, ^27^ Regarding date of publication, the most recently published studies reported higher proportions of GI symptoms, which were statistically significant for diarrhea (after 1 April, 16%, 95% CI 13–20%; on or before 1 April, 9%, 95% CI 7–12%, Figure S16), nausea or vomiting (after 1 April, 12%, 95% CI 8–16%; on or before 1 April, 6%, 95% CI 4–9%, Figure S17), and any GI symptoms (after 1 April, 24%, 95% CI 18–34%; on or before 1 April, 13%, 95% CI 9–19%, Figure S18), but not for abdominal pain or anorexia, though the effects were all in the same direction (Figures S19–20). Some of the effect of geography and publication date covaried, in that the majority of earlier published studies were from China. In a logit multivariable meta‐regression model incorporating both variables, publication date was no longer statistically significant for any of the GI symptoms (*P* = 0.14 for diarrhea, *P* = 0.72 for nausea/vomiting), though retained the same direction of effect (Diarrhea 14%, 95% CI 10–17%, after 1 April; 10%, 95% CI 9–13% before 1 April; Nausea/vomiting 8%, 95% CI 6–12% after 1 April; 8%, 95% CI 5–11% before 1 April). Asian studies still reported statistically significant lower rates of diarrhea and nausea or vomiting, however, even when adjusted for publication date (*P* = 0.02 and 0.01 respectively). There were no significant differences in the proportions of patients with any GI symptom according to the other study characteristics evaluated (eResults).

### Prevalence of hepatic inflammation

**Figure 3:**
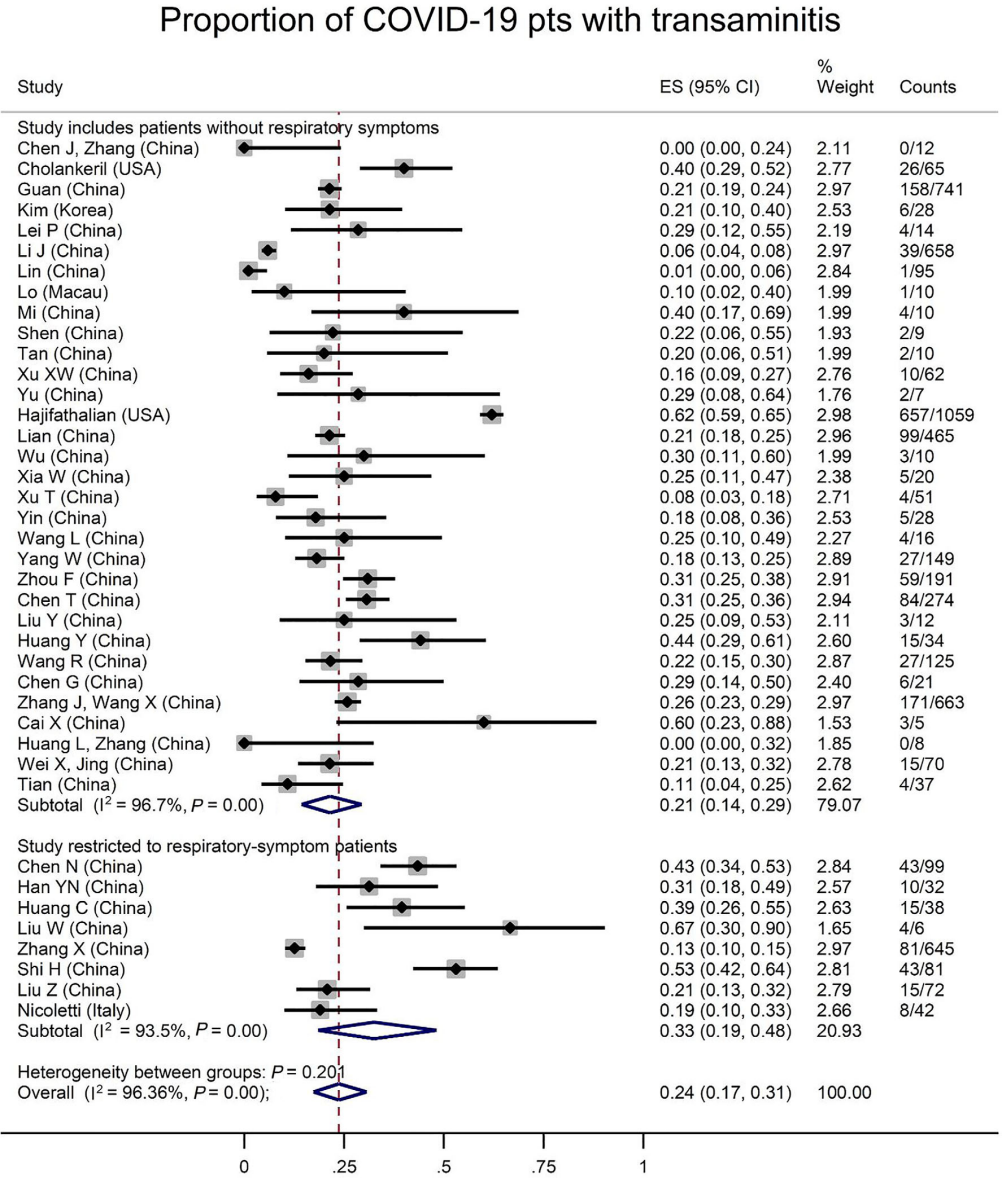
Proportion of all coronavirus disease (COVID)‐19 patients with elevated transaminases, by studies restricted to patients with respiratory symptoms or not. Overall prevalence of elevated transaminases in COVID‐19 infection was 24% (95% confidence interval 17–31%). This proportion was 33% in studies restricted to patients with respiratory symptoms, and only 21% in studies including patients without respiratory symptoms (*P* = 0.20).

Sixty‐two studies reported liver enzymes on some participants, with an overall prevalence of an abnormal transaminase in 24% of patients with COVID‐19 (95% CI 17–31%, I^2^ 96%, Fig. 3). Similar to luminal symptoms, Asian studies reported lower proportions of elevated transaminases, although there was no significant difference in liver injury by study publication date (Figures S21–22). The average ALT was 34.8 (±16.1) units per liter (U/L), and AST 39.0 U/L (±17.3) among all COVID‐19 patients, from 44 and 43 studies, respectively. The weighted average AST:ALT ratio was 1.15 (±0.20). When restricted to only those patients with abnormal levels, average ALT was 48.7 U/L (±22.4) and AST 47.3 U/L (±23.9), although the weighted average AST:ALT ratio from each of the 11 studies reporting mean AST and ALT values in this subgroup was 1.06 (±0.32).

### Clinical outcomes

**Figure 4:**
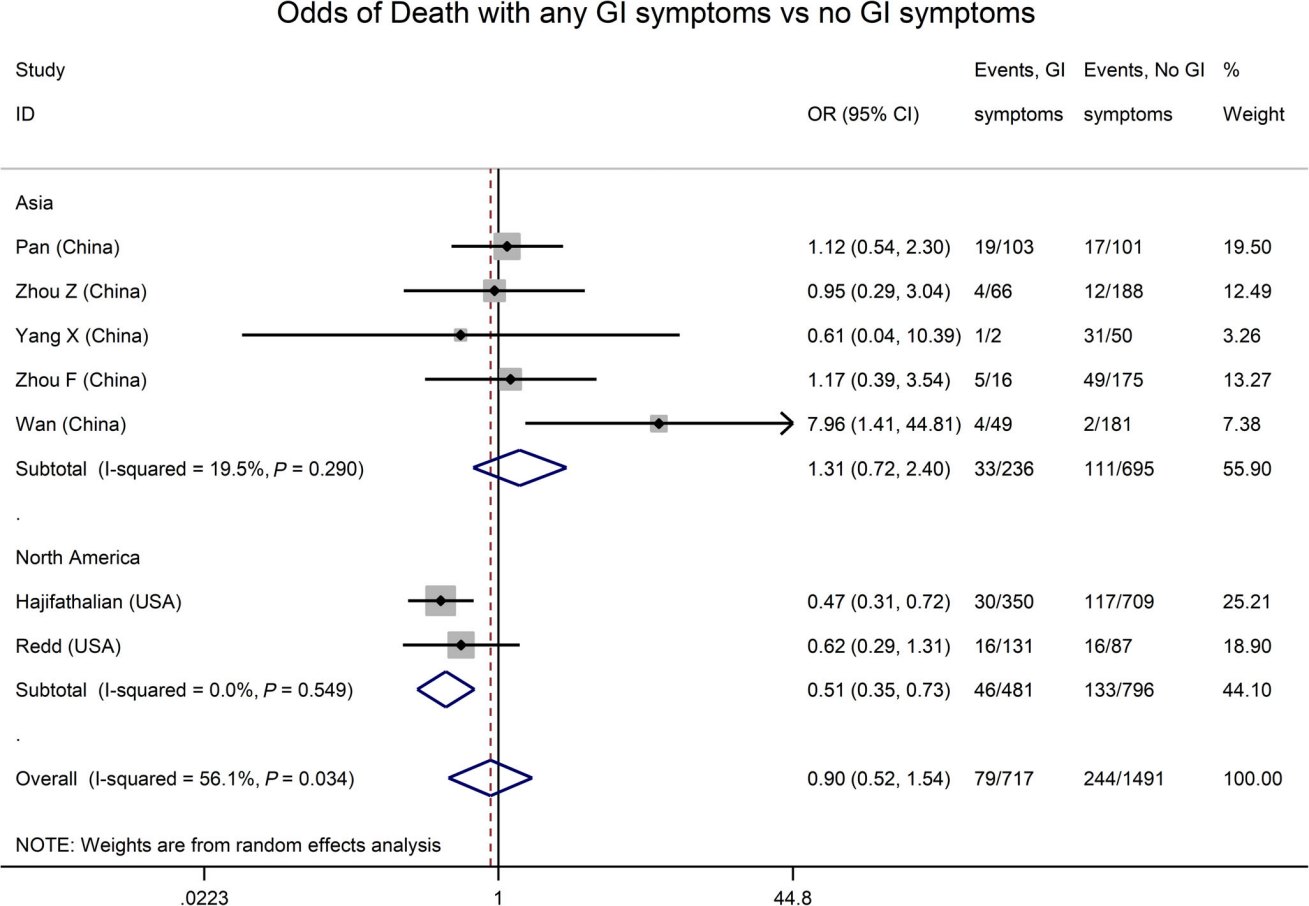
Odds of mortality in coronavirus disease (COVID‐19) infection with gastrointestinal (GI) symptoms, by continent. Odds of mortality were not significantly different (odds ratio [OR] 0.90, 95% confidence interval [CI] 0.52–1.54) in COVID‐infected patients with GI symptoms compared to those without. In the two US studies, however, the pooled odds ratio for mortality with GI symptoms was 0.51 (95% CI 0.35–0.73),*versus*OR 1.31 (95% CI 0.72–2.40) in studies from China (*P* = 0.04 for difference).

There was a mix of clinical severity among the studies, with 971/11 943 of overall patients experiencing mortality (75 studies), 489/8390 mechanical ventilation (59 studies), and 697/8257 intensive care unit (ICU) admission (62 studies). Random‐effects for the proportions of these outcomes from studies that included hospitalized patients of any severity were 3% (95% CI 1–5%), 4% (95% CI 2–5%), and 6% (95% CI 4–9%), respectively. From seven studies that reported these clinical outcomes according to the presence of GI symptoms,^2^, ^10^, ^17^, ^28^, ^29^, ^30^, ^31^ odds of death was not significantly different based on the presence of GI symptoms (OR 0.90, 95% CI 0.52–1.54, Fig. 4). Exclusion of a single outlier study where GI symptoms were also associated with older age^31^ revealed a significant negative association of GI symptoms with mortality (OR 0.68, 95% CI 0.48–0.97), although this was driven by two large North American studies (Fig. 4).^29^, ^30^

A largely non‐overlapping group of 12 studies,^14^, ^32^, ^33^, ^34^, ^35^, ^36^, ^37^, ^38^, ^39^, ^40^ all from China, reported on the presence of severe *versus* non‐severe COVID‐19^41^ in association with GI manifestations. Overall, patients with any GI symptoms had greater odds of severe COVID‐19 related disease than those without GI symptoms (OR 2.07, 95% CI 1.34–3.18, I^2^ 41%, Figure S23). Several outliers were very small studies (5 with <5 subjects with GI symptoms), but excluding these did not affect the point estimate or the significance of the result (OR 2.18, 95% CI 1.38–3.45, I^2^ 56%). The associations of severe COVID‐19 with individual symptoms were in the same direction, although not statistically significant (Figures S24–26).

## Discussion

This meta‐analysis represents the largest study of gastrointestinal manifestations of COVID‐19 to date, with over 17 000 patients from 108 studies. Overall, GI symptoms are common in COVID‐19, and highest in publications from outside of China and after 1 April, with one in four patients reporting some GI symptom, including 16% with diarrhea and 12% with nausea or vomiting in the most recent studies. One quarter of patients also demonstrate transaminase elevations. Presentation with GI symptoms without any respiratory symptoms or fever is uncommon but remains clinically relevant for a pandemic of this magnitude, occurring in 1% of patients.

Our primary analysis demonstrating that 1% (95% CI 0–6%) of COVID‐19 patients present with isolated GI symptoms (up to 2% if presence of fever allowed) is lower than reported in previous studies. For example, an earlier review by Mao *et al*. reported that 10% of COVID‐19 patients presented with only GI symptoms, and a matched cohort study from a single hospital in Wuhan by Han *et al*. reported isolated GI symptoms in 23% of COVID‐19 patients.^3^, ^42^ However, the Han *et al*. study was not able to estimate a true prevalence of GI symptoms among all COVID‐19 patients. Furthermore, our review identifies and includes several studies with zero prevalence of isolated GI symptoms that were not included in the Mao review. Even if the overall prevalence of isolated GI symptoms is relatively low, 1% represents hundreds of cases daily in the US alone, with both clinical and public health implications.^43^, ^44^

Our study differentiates between GI symptoms with and without anorexia, with the reported prevalence of GI symptoms varying from 28% when anorexia was included to 17% when excluding this symptom. Anorexia may be the result of systemic inflammation rather than a true digestive symptom.^45^ The inclusion of this non‐specific infectious symptom may artificially elevate the prevalence of GI symptoms reported in some studies,^2^, ^46^ a distinction the clinician should keep in mind when interpreting this literature.

In exploring the significant heterogeneity inherent in a review of large numbers of studies, geography and publication date emerged as significantly associated with reported prevalence of GI symptoms. The prevalence of diarrhea and nausea/vomiting in publications printed after 1 April was nearly twice that of publications before 1 April and in publications from Europe or North America compared to Asia. Given that earlier cases and publications occurred in Asia, the effect of country *versus* publication date is challenging to disentangle. As COVID‐19 was first described as a respiratory illness, comprehensive collection of symptom data (by investigators as well as the clinicians populating the medical records) occurred to a lesser extent earlier in the pandemic. The results of the largest studies from our review are illustrative. Guan *et al*. (February 2020) reported 3.8% of patients had diarrhea and 5% nausea and/or vomiting. However, study investigators reported incomplete documentation of disease course due to overwhelmed medical infrastructure and clinically—rather than research‐driven data collection.^47^ Cholankeril *et al*. (April 2020) reported higher prevalences of diarrhea (12%) and nausea/vomiting (12%), but the authors acknowledged testing criteria had not included extrapulmonary symptoms at the time.^48^ In contrast, Hajifathalian (New York study from May) clearly defined GI symptoms and systematically evaluated patients for extrapulmonary manifestations. They reported even higher prevalence of diarrhea (22%).^29^ We suggest that the pooled prevalence from the most recent studies (diarrhea 16%, 95% CI 13–20%; nausea/vomiting 12%, 95% CI 8–16%) are therefore the most accurate estimates of the true prevalence of GI symptoms in SARS‐CoV2.

We found liver inflammation to be even more prevalent than GI symptoms with 24% of patients having elevated transaminases. The mechanism of liver injury in COVID‐19 patients is not yet well understood. While angiotensin‐converting enzyme 2 (ACE 2) receptors exist on bile duct epithelium and hepatocytes, liver injury is more likely due to immune activation^49^, ^50^ than direct viral cytopathic effect.^51^, ^52^ Many cases of hepatic dysfunction in severe COVID‐19 are likely multifactorial, including insults from hypoxia, hypoperfusion, thrombosis, and hepatotoxic medications. The average transaminase level even in patients with abnormal levels was not severely elevated (<50 U/L), which suggests that liver inflammation is mild and not a major part of COVID‐19 related inflammation. Finally, synthetic liver dysfunction clearly linked to COVID‐19 infection has yet to be described.^53^

This review also examined the relationship between GI symptoms and clinical outcomes in COVID‐19, with mixed results. Pooled data from 12 Chinese studies yielded an association between GI symptoms and severe COVID‐19, similar to prior reviews.^42^, ^54^ However, patients with GI symptoms showed a trend toward lower mortality. The main reason for this apparent inconsistency is likely the different sets of studies reporting each outcome, as the inverse relationship with mortality was driven by two large US studies,^29^, ^54^ also published after prior reviews.^42^ Regardless, the association with severe disease is limited by heterogeneous definitions of what constitutes “severe,” and possibly other factors such as late‐onset GI symptoms resulting from treatments in patients that are more ill. The small number of studies evaluating the association of GI symptoms with outcomes indicate that more research is needed in this area.

Our meta‐analysis has notable limitations. As a review of a large number of observational studies, there is a great degree of heterogeneity, which is only partially explained by subgroup analyses. The prevalence estimates of less variably defined outcomes—such as isolated GI symptoms, diarrhea, and vomiting—are therefore more reliable than the outcomes of “any” GI symptoms or anorexia. The majority of included studies are Chinese and from hospitalized settings, limiting the generalizability of these findings to other regions and less severely ill patients. Lastly, no studies had low risk of bias. This rating was usually due to lack of COVID‐19 negative comparison groups, suboptimal measurement of GI symptoms, and inadequate follow‐up of COVID‐19 patients. In fact, recent articles have drawn attention to the poor methodology of many early COVID‐19 studies, driven by overwhelmed healthcare systems in need of rapid information dissemination with abbreviated peer review.^55^, ^56^ These limitations notwithstanding, our review is the largest to‐date of the peer‐reviewed literature, including separate subgroup analyses of literature published since 1 April, to provide the most updated estimates of the prevalence of GI and hepatic effects of COVID‐19 infection.

We advocate broad testing for COVID‐19—including for patients presenting with isolated GI symptoms or unexplained elevations of LFTs—when testing resources are plentiful. While COVID‐19 with isolated GI symptoms may not be common enough (1–2%) to warrant testing on this basis alone when testing resources are scarce, these patients should consider self‐quarantine to monitor for development of other symptoms, including fever, cough, and dyspnea. The significant role of digestive symptoms in COVID‐19 is clear, but many knowledge gaps regarding their pathophysiology and prognostic value remain. Further research should proceed through well‐designed prospective studies.

## Supporting information


**Appendix S1.** Supporting information.
