# Supplementary material for: Gastrointestinal and hepatic manifestations of COVID‐19: A systematic review and meta‐analysis
Source: JGH Open. 2020 Nov 21;5(1):107–15. doi: 10.1002/jgh3.12456 (PMC7753450; doi:10.1002/jgh3.12456)
Supplement: Supplementary file 1 — Appendix S1. Supporting information. [file JGH3-5-107-s001.docx]

Online-Only Supplement

eMethods. Additional description of study selection, data extraction, and quantitative analysis.

eResults. Description of additional subgroup analyses.

eTable 1. Characteristics of included studies.

eTable 2. Risk of bias assessments for included studies.

**eFigure 1**. Proportion of COVID-19 patients with any GI symptom.

**eFigure 2.** Proportion of COVID-19 patients with GI symptoms as part of initial presentation.

**eFigure 3.** Proportion of COVID-19 patients with only GI symptoms, regardless of definition of fever.

**eFigure 4.** Proportion of COVID-19 patients with only GI symptoms, without fever.

**eFigure 5**. Funnel plot of proportion of COVID-19 patients with only GI symptoms (by standard error of proportion).

**eFigure 6.** Proportion of all COVID-19 patients with diarrhea, by whether cohort restricted to respiratory symptoms or not.

**eFigure 7.** Proportion of all COVID-19 patients with nausea and/or vomiting.

**eFigure 8.** Proportion of all COVID-19 patients with vomiting.

**eFigure 9**. Proportion of all COVID-19 patients with abdominal pain.

**eFigure 10.** Proportion of all COVID-19 patients with anorexia / loss of appetite.

**eFigure 11.** Proportion of all COVID-19 patients with diarrhea, by continent.

**eFigure 12**. Proportion of all COVID-19 patients with nausea or vomiting, by continent.

**eFigure 13.** Proportion of all COVID patients with abdominal pain, by continent.

**eFigure 14.** Proportion of all COVID-19 patients with any GI symptoms, by continent.

**eFigure 15.** Proportion of all COVID-19 patients with anorexia, by continent.

**eFigure 16.** Proportion of all COVID-19 patients with diarrhea, by publication date.

**eFigure 17.** Proportion of all COVID-19 patients with nausea or vomiting, by publication date.

**eFigure 18.** Proportion of all COVID-19 patients with anorexia, by publication date.

**eFigure 19.** Proportion of all COVID-19 patients with abdominal pain, by publication date.

**eFigure 20.** Proportion of patients with any GI symptoms, by publication date.

**eFigure 21.** Proportion of all COVID-19 patients with elevated transaminases, by continent.

**eFigure 22.** Proportion of all COVID-19 patients with elevated transaminases, by publication date.

**eFigure 23**. Odds of severe COVID-19 in patients with GI symptoms compared to those without.

**eFigure 24.** Odds of severe COVID-19 in patients with diarrhea.

**eFigure 25.** Odds of severe COVID-19 in patients with nausea or vomiting.

**eFigure 26.** Odds of severe COVID-19 in patients with abdominal pain.

**eMethods.**

*Search Strategy*

Pubmed, EMBASE, and Cochrane search terms included below:

PubMed Strategy

| 1. | (“Coronavirus” [Mesh] OR “Coronavirus Infections” [Mesh] OR “COVID-19” [Supplementary Concept] OR “severe acute respiratory syndrome coronavirus 2” [Supplementary Concept]) OR Wuhan [tiab] OR Coronavirus [tiab] OR “Corona virus” [tiab] OR Covid [tiab] OR nCov [tiab] OR “novel corona virus” [tiab] OR “novel coronavirus” [tiab] OR “covid 19” [tiab] OR “covid-19” [tiab] OR “Covid19” [tiab] OR “SARS-CoV-2” [tiab] OR SARSCoV2 [tiab] OR “severe acute respiratory syndrome coronavirus 2” [tiab] | 47,569 |
| --- | --- | --- |
| 2. | Vomiting [mesh] OR nausea [mesh] OR diarrhea [mesh] OR gastroenteritis [mesh:noexp] OR dysentery [mesh] OR anorexia [mesh] OR appetite [mesh] OR appetite regulation [mesh] OR abdominal pain [mesh] OR Vomiting [tiab] OR vomit [tiab] OR vomits [tiab] OR vomited [tiab] OR emesis [tiab] OR emeses [tiab] OR emetic [tiab] OR nausea [tiab] OR nauseous [tiab] OR nauseate [tiab] OR nauseates [tiab] OR nauseated [tiab] OR queasy [tiab] OR queasiness [tiab] OR hyperemesis [tiab] OR hyperemetic [tiab] OR retch [tiab] OR retches [tiab] OR retched [tiab] OR retching [tiab] OR dyspepsia [tiab] OR dyspeptic [tiab] OR diarrhea [tiab] OR diarrheal [tiab] OR dysentery [tiab] OR “loose stool” [tiab] OR gastrointestinal [tiab] OR anorexia [tiab] OR anorectic [tiab] OR anorexic [tiab] OR appetite [tiab] OR satiety [tiab] OR satiation [tiab] OR hunger [tiab] OR hungry [tiab] OR fullness [tiab] OR “food behavior” [tiab] OR “food behaviour” [tiab] OR “eating behavior” [tiab] OR “eating behaviour” [tiab] OR “food intake” [tiab] OR “dietary intake” [tiab] OR (("Stomach"[Mesh] OR "Abdomen"[Mesh] OR “Intestines” [mesh] OR Stomach [tiab] OR Abdomen [tiab] OR abdominal [tiab] OR Intestine [tiab] OR intestines [tiab] OR intestinal [tiab] OR Bowel [tiab] OR Belly [tiab] OR Gastrointestinal [tiab] OR Gastric [tiab] OR gut [tiab] OR viscera [tiab] OR visceral [tiab]) AND (Pain [tiab] OR pains [tiab] OR Distress [tiab] OR Discomfort [tiab] OR ache [tiab] OR aches [tiab] OR sore [tiab] OR soreness [tiab] OR cramp [tiab] OR cramps [tiab] OR cramping [tiab])) | 677,774 |
| 3. | "Liver Diseases" [MESH] OR liver [tiab] OR hepatic [tiab] OR "transaminases" [MeSH Terms] OR "transaminases" [tw] OR "transaminase" [tw] OR Aspartate Aminotransferases [tw] OR Cytoplasmic Aspartate Aminotransferase [tw] OR Mitochondrial Aspartate Aminotransferase [tw] OR Hypertransaminasemia [tw] OR transaminitis [tw] | 1,205,458 |
| 4. | 2019/12/01[PDAT]:2020/05/18[PDAT] | 768,784 |
| 5. | English [la] | 26,445,496 |
|  |  |  |
| 6. | #1 and #2 and #3 and #4 and #5 (covid/gastro/liver/pubdate/English) | 46 |
|  |  |  |
| 7. | #1 and #3 and #4 and #5 (covid/liver/pubdate/English) | 248 |
| 8. | #1 and #2 and #4 and #5 (covid/gastro/pubdate/English) | 399 |

Cochrane Strategy

| 1 | [mh “Coronavirus”] OR [mh “Coronavirus Infections”] OR Wuhan:ti,ab,kw OR Coronavirus:ti,ab,kw OR “Corona virus”:ti,ab,kw OR Covid:ti,ab,kw OR nCov:ti,ab,kw OR “novel corona virus”:ti,ab,kw OR “novel coronavirus”:ti,ab,kw OR “covid 19”:ti,ab,kw OR “covid-19”:ti,ab,kw OR “Covid19”:ti,ab,kw OR “SARS-CoV-2”:ti,ab,kw OR SARSCoV2:ti,ab,kw OR “severe acute respiratory syndrome coronavirus 2”:ti,ab,kw | 883 |
| --- | --- | --- |
| 2 | [mh “Vomiting”] OR [mh “nausea”] OR [mh “diarrhea”] OR [mh “gastroenteritis”] OR [mh “dysentery”] OR [mh “anorexia”] OR [mh “appetite”] OR [mh “appetite regulation”] OR [mh “abdominal pain”] OR Vomit*:ti,ab,kw OR emesis:ti,ab,kw OR emeses:ti,ab,kw OR emetic:ti,ab,kw OR nause*:ti,ab,kw OR queasy:ti,ab,kw OR queasiness:ti,ab,kw OR hyperemesis:ti,ab,kw OR hyperemetic:ti,ab,kw OR retch*:ti,ab,kw OR dyspepsia:ti,ab,kw OR dyspeptic:ti,ab,kw OR diarrhea*:ti,ab,kw OR dysentery:ti,ab,kw OR “loose stool”:ti,ab,kw OR gastrointestinal:ti,ab,kw OR anorexia:ti,ab,kw OR anorectic:ti,ab,kw OR anorexic:ti,ab,kw OR appetite:ti,ab,kw OR satiety:ti,ab,kw OR satiation:ti,ab,kw OR hunger:ti,ab,kw OR hungry:ti,ab,kw OR fullness:ti,ab,kw OR “food behavior”:ti,ab,kw OR “food behaviour”:ti,ab,kw OR “eating behavior”:ti,ab,kw OR “eating behaviour”:ti,ab,kw OR “food intake”:ti,ab,kw OR “dietary intake”:ti,ab,kw OR ((Stomach:ti,ab,kw OR Abdomen:ti,ab,kw OR abdominal:ti,ab,kw OR Intestin*:ti,ab,kw OR Bowel*:ti,ab,kw OR Belly:ti,ab,kw OR Gastrointestinal:ti,ab,kw OR Gastric:ti,ab,kw OR gut:ti,ab,kw OR viscera*:ti,ab,kw) AND (Pain:ti,ab,kw OR pains:ti,ab,kw OR Distress:ti,ab,kw OR Discomfort:ti,ab,kw OR ache:ti,ab,kw OR aches:ti,ab,kw OR sore:ti,ab,kw OR soreness:ti,ab,kw OR cramp*:ti,ab,kw)) | 140,978 |
| 3 | [mh "Liver Diseases"] OR liver:ti,ab,kw OR hepatic:ti,ab,kw OR [mh "transaminases"] OR "transaminases":ti,ab,kw OR "transaminase":ti,ab,kw OR Aspartate Aminotransferases:ti,ab,kw OR Cytoplasmic Aspartate Aminotransferase:ti,ab,kw OR Mitochondrial Aspartate Aminotransferase:ti,ab,kw OR Hypertransaminasemia:ti,ab,kw OR transaminitis:ti,ab,kw | 62255 |
| 4 | #2 or #3 | 191,791 |
| 5 | #1 and #4 | 52 |

Embase Strategy

| 1. | ‘Coronavirinae’/exp OR ‘Coronavirus Infection’/exp OR ‘COVID 19’/exp OR ‘coronavirus disease 2019’/exp OR ‘severe acute respiratory syndrome coronavirus 2’/exp OR Wuhan:ab,ti OR Coronavirus:ab,ti OR “Corona virus”:ab,ti OR Covid:ab,ti OR nCov:ab,ti OR “novel corona virus”:ab,ti OR “novel coronavirus”:ab,ti OR “covid 19”:ab,ti OR “Covid19”:ab,ti OR “SARS CoV 2”:ab,ti OR SARSCoV2:ab,ti OR “severe acute respiratory syndrome coronavirus 2”:ab,ti | 49,482 |
| --- | --- | --- |
| 2. | ‘nausea and vomiting’/exp OR ‘diarrhea’/exp OR ‘gastroenteritis’/exp OR ‘dysentery’/exp OR ‘anorexia’/exp OR ‘appetite’/exp OR ‘food intake’/exp OR ‘abdominal pain’/exp OR Vomiting:ab,ti OR vomit:ab,ti OR vomits:ab,ti OR vomited:ab,ti OR emesis:ab,ti OR emeses:ab,ti OR emetic:ab,ti OR nausea:ab,ti OR nauseous:ab,ti OR nauseate:ab,ti OR nauseates:ab,ti OR nauseated:ab,ti OR queasy:ab,ti OR queasiness:ab,ti OR hyperemesis:ab,ti OR hyperemetic:ab,ti OR retch:ab,ti OR retches:ab,ti OR retched:ab,ti OR retching:ab,ti OR dyspepsia:ab,ti OR dyspeptic:ab,ti OR diarrhea:ab,ti OR diarrheal:ab,ti OR dysentery:ab,ti OR “loose stool”:ab,ti OR gastrointestinal:ab,ti OR anorexia:ab,ti OR anorectic:ab,ti OR anorexic:ab,ti OR appetite:ab,ti OR satiety:ab,ti OR satiation:ab,ti OR hunger:ab,ti OR hungry:ab,ti OR fullness:ab,ti OR “food behavior”:ab,ti OR “food behaviour”:ab,ti OR “eating behavior”:ab,ti OR “eating behaviour”:ab,ti OR “food intake”:ab,ti OR “dietary intake”:ab,ti OR ((‘stomach’/exp OR ‘abdomen’/exp OR ‘intestine’/exp OR Stomach:ab,ti OR Abdomen:ab,ti OR abdominal:ab,ti OR Intestine:ab,ti OR intestines:ab,ti OR intestinal:ab,ti OR Bowel:ab,ti OR Belly:ab,ti OR Gastrointestinal:ab,ti OR Gastric:ab,ti OR gut:ab,ti OR viscera:ab,ti OR visceral:ab,ti) AND (Pain:ab,ti OR pains:ab,ti OR Distress:ab,ti OR Discomfort:ab,ti OR ache:ab,ti OR aches:ab,ti OR sore:ab,ti OR soreness:ab,ti OR cramp:ab,ti OR cramps:ab,ti OR cramping:ab,ti)) | 1,501,620 |
| 3 | 'liver disease'/exp OR liver:ab,ti OR 'aminotransferase'/exp OR aminotransferase:ab,ti OR transaminase:ab,ti OR transaminases:ab,ti OR aminotransferases:ab,ti OR hypertransaminasemia:ab,ti OR transaminitis:ab,ti | 1,702,276 |
|  |  |  |
| 4 | **[2019-2020]/py** | 2,381,076 |
|  |  |  |
| 5. | [English]/lim | 31,385,730 |
|  |  |  |
| 6. | #1 and #2 and #3 and #4 and #5 | 151 |
|  |  |  |
| 7. | #1 and #3 and #4 and #5 | 697 |

*Study selection and data abstraction*

We excluded unrefereed preprints, meeting abstracts, and studies not available in English. Reference lists of relevant review articles were hand searched for studies missed in the electronic search. All study designs were included inasmuch as they permitted calculation of the incidence of GI symptoms in a COVID-19 positive cohort, therefore excluding most studies defined as case reports or case series. We included both pediatric and adult studies. Two reviewers (EB, AL) independently reviewed all studies for inclusion based on title/abstract, with disagreements eligible for full text review. EB and AL also reviewed the full texts, with dissenting eligibility resolved by consensus and input from a third reviewer (RD).

Data extracted included study location, number of COVID-19 positive patients, method of testing and cohort identification, patient sample demographics, presence of comorbid digestive and liver disease, number of subjects with gastrointestinal symptoms with and without fever or systemic symptoms, and method of symptom assessment. We also recorded clinical outcomes, including severe versus non-severe COVID-19, death, ICU admission, and mechanical ventilation, stratified by presence of GI symptoms when possible. Severe COVID-19 was defined by each study. AL and EB independently extracted the data, and RD reviewed all extracted data for accuracy.

ROB was assessed for the primary outcome of prevalence of GI symptoms using the Newcastle Ottawa Scale, and categorized based on numerical score into low (>7), medium (4-6), or high (<4) risk of bias.

*Quantitative analysis*

Transaminase elevation was defined according to each study’s laboratory reference range, The Freeman-Tukey double arcsine transformation was selected for meta-analysis of proportions, allowing inclusion of studies with proportions at or near zero without use of continuity correction, and without undue weighting or 95% confidence intervals of the individual studies that cross the null. Sensitivity analyses were performed using the logit transformation and the default procedure in *metan* using confidence intervals from the normal distribution based on the asymptotic variance. These statistics did not vary greatly from the Freeman-Tukey transformation, but excluded progressively greater numbers of studies. For analyses of small study effects, we plotted the incidence of GI symptoms versus the standard error of the Freeman Tukey-transformed proportion, as well as a sensitivity analysis versus sample size. Stata packages used included *metaprop_one* and *metapreg* for proportions, *metan* for odds ratios, *metafunnel* and *metabias* for small study effects.

**eResults**

*Study characteristics*

Overall 50.4% (+/-9.2%) of subjects were male and the average age of patients in adult-only studies was 53.3 (+/-6.8). The main components leading to risk of bias related to cohort selection and outcome ascertainment.

*Incidence of Luminal GI Symptoms, Subgroup Analyses*

In multivariable meta-regression for geography and publication date, adjusted incidence of diarrhea was 11% (95%CI 9-13%) in Asian studies and 20% (95%CI 13-31%) outside of Asia, while nausea/vomiting was 7% (95%CI 5-9%) prevalent in Asian studies and 16% (95%CI 9-27%) outside of Asia, and any GI symptoms 16% prevalent (95%CI 12-21%) in Asian studies and 26% (95%CI 15-41%) in studies outside of Asia. There were no significant differences in the proportions of patients with any GI symptom according to study design (prospective vs retrospective), manuscript type (full length, short communication, or letter to the editor), risk of bias rating (medium or high), paediatric vs adult, study setting (hospital or clinic), or COVID case definition (upper respiratory PCR vs other). There were no significant subgroup effects (including by geography or publication date) observed in the analysis of patients with only GI symptoms, due to the fewer studies, lower rates, and less heterogeneity with this outcome. There was no evidence of funnel plot asymmetry for the proportion of subjects with any of the individual GI symptoms, or transaminase elevation.

**eTable 1.** Characteristics of included studies.

| Author | Location | Date published | Study Patient Subset | #Covid Patients | Average age | %male | AnyGI | GI First Symptom | Diarrhea | Anorexia | OnlyGI no fever | Elevated LFTs | Severe COVID |
| --- | --- | --- | --- | --- | --- | --- | --- | --- | --- | --- | --- | --- | --- |
| Cai X ^1^ | China | 5/12/2020 | N/A | 5 | 1.7 | 80 | 4 | 4 | 2 | 2 | 1 | 3 | 2 |
| Chang^2^ | China | 2/7/2020 | N/A | 13 | 34 | 77 | 1 | N/A | 1 | N/A | N/A | N/A | 0 |
| Chen G ^3^ | China | 3/27/2020 | N/A | 21 | 56 | 81 | 4 | N/A | 4 | N/A | N/A | 6 | 11 |
| Chen J ^4^ | China | 4/14/2020 | N/A | 12 | 14.5 | 50 | 4 | 0 | 4 | N/A | N/A | 0 | 0 |
| Chen J^5^ | China | 3/19/2020 | N/A | 249 | 51 | 50.6 | N/A | N/A | 8 | 8 | N/A | N/A | N/A |
| Chen M^6^ | China | 5/13/2020 | Patients who were Re-hospitalized | 11 | 48.45 | 73 | 5 | N/A | 2 | N/A | N/A | N/A | N/A |
| Chen N^7^ | China | 2/15/2020 | Patients with COVID Pneumonia | 99 | 55.5 | 68 | N/A | N/A | 2 | N/A | N/A |  | N/A |
| Chen Q^8^ | China | 3/12/2020 | N/A | 9 | 42.1 | 55.5 | 2 | N/A | 2 | N/A | N/A | N/A | 4 |
| Chen Q^9^ | China | 4/28/2020 | N/A | 145 | 47.5 | 54.5 | N/A | N/A | 39 | 62 | N/A | N/A | 43 |
| Chen T^10^ | China | 3/26/2020 | Severely ill patients | 274 | 62 | 62 | N/A | N/A | 77 | 66 | N/A | 84 | 274 |
| Chen Y^11^ | China | 4/3/2020 | N/A | 42 | 51 | 35.7 | 8 | N/A | 7 | N/A | N/A | N/A | 11 |
| Cholankeril^12^ | USA | 4/10/2020 | N/A | 116 | 50 | 53.4 | 37 | 0 | 12 | 22 | N/A | 26 | N/A |
| Dai^13^ | China | 4/1/2020 | Patients with CT Results Obtained | 234 | 44.6 | 58.1 | N/A | N/A | 9 | N/A | N/A | N/A | 15 |
| Diao^14^ | China | 3/1/2020 | Patients with CT Results Obtained | 6 | 42.7 | 50 | 1 | N/A | 1 | N/A | N/A | N/A | 0 |
| Effenberger^15^ | Austria | 4/20/2020 | N/A | 40 | 65.445 | 60 | N/A | N/A | 22 | N/A | N/A | N/A | N/A |
| Garazzino^16^ | Italy | 5/7/2020 | N/A | 168 | 5 | 55.9 | N/A | N/A | 22 | N/A | N/A | N/A | N/A |
| Guan^17^ | China | 2/28/2020 | N/A | 1099 | 47 | 58.1 | N/A | N/A | 42 | N/A | N/A | 158 | 173 |
| Hajifathalian^18^ | USA | 5/7/2020 | N/A | 1059 | 61.1 | 57.7 | 350 | N/A | 234 | 240 | N/A | 657 | N/A |
| Han C^19^ | China | 4/15/2020 | Patients with GI Symptoms | 206 | 62.5 | 44.2 | 117 | 13 | 67 | 102 | N/A | N/A | 0 |
| Han R^20^ | China | 3/17/2020 | Patients with COVID Pneumonia | 108 | 45 | 35.2 | 15 | N/A | 15 | N/A | N/A | N/A | N/A |
| Han YN^21^ | China | 4/6/2020 | Patients with Acute Respiratory Illness | 32 | 44 | 52 | 6 | N/A | N/A | N/A | N/A | 10 | 4 |
| He^22^ | China | 4/12/2020 | Patients with COVID Pneumonia | 204 | 49 | 38.73 | N/A | N/A | 19 | 12 | N/A | N/A | 69 |
| Hossain^23^ | USA | 5/11/2020 | Patients with Gastrointe-stinal or Neurologic Complaints | 119 | 65 | 49.6 | 60 | N/A | N/A | N/A | N/A | N/A | 29 |
| Huang C^24^ | China | 1/30/2020 | Patients with COVID Pneumonia | 38 | 49 | 73 | 1 | N/A | 1 | N/A | N/A | 15 | 13 |
| Huang L^25^ | China | 4/14/2020 | Familial cluster | 7 | 33.4 | 28.5 | 1 | 1 | 1 | N/A | 1 | N/A | N/A |
| Huang LE ^26^ | China | 4/10/2020 | N/A | 8 | 22 | 75% | 7 | 0 | 6 | 3 | 0 | 0 | 0 |
| Huang Y^27^ | China | 2/27/2020 | N/A | 34 | 56.24 | 41.2 | 5 | N/A | 5 | N/A | N/A | 15 | N/A |
| Jin^28^ | China | 3/24/2020 | N/A | 651 | 45 | 50.8 | 74 | N/A | 56 | N/A | N/A | N/A | 64 |
| Kim^29^ | Korea | 4/6/2020 | N/A | 28 | 40 | 53.6 | N/A | N/A | 14 | N/A | N/A | 6 | 6 |
| Klopfenstein^30^ | France | 4/27/2020 | N/A | 114 | 56 | 42 | 55 | N/A | 55 | N/A | N/A | N/A | 4 |
| Lechien^31^ | Europe | 4/30/2020 | Patients with Mild to Moderate Disease | 1420 | 39.17 | 32.3 | N/A | N/A | 473 | 649 | N/A | N/A | 0 |
| Lei P^32^ | China | 4/7/2020 | N/A | 14 | 47 | 57 | 3 | N/A | 3 | N/A | 0 | 4 | N/A |
| Lei Z^33^ | China | 4/9/2020 | Non-Critically Ill Patients | 20 | 43.2 | 50 | N/A | N/A | 5 | N/A | N/A | N/A | N/A |
| Li^34^ | China | 4/20/2020 | N/A | 658 | 57.5 | 45.1 | N/A | N/A | 18 | N/A | N/A | 39 | N/A |
| Li^35^ | China | 6/1/2020 | Patients with Respiratory Symptoms who had a CT | 83 | 45.5 | 53 | 7 | N/A | N/A | N/A | N/A | N/A | 25 |
| Lian^36^ | China | 5/12/2020 | N/A | 465 | 45 | 52.26 | N/A | N/A | 36 | N/A | N/A | 99 | 49 |
| Lin^37^ | China | 4/2/2020 | N/A | 95 | 45.3 | 47.4 | 11 | N/A | 5 | 5 | N/A | 1 | 20 |
| Liu F^38^ | China | 3/6/2020 | N/A | 10 | 42 | 40 | 3 | N/A | 0 | N/A | 0 | N/A | 5 |
| Liu J^39^ | China | 4/18/2020 | N/A | 40 | 48.7 | 37.5 | N/A | N/A | 3 | N/A | N/A | N/A | 13 |
| Liu J-Y^40^ | Taiwan | 5/9/2020 | N/A | 321 | N/A | 47 | 26 | N/A | 23 | N/A | N/A | N/A | N/A |
| Liu K^41^ | China | 2/7/2020 | Patients with Respiratory Symptoms | 137 | 57 | 44.5 | 11 | N/A | 11 | N/A | N/A | N/A | N/A |
| Liu W^42^ | China | 3/12/2020 | Patients with Respiratory Infections | 6 | 6 | N/A | 4 | N/A | N/A | N/A | 0 | 4 | 1 |
| Liu Y^43^ | China | 2/9/2020 | N/A | 12 | 53.7 | 66.7 | 3 | N/A | 2 | N/A | 0 | 3 | 5 |
| Liu Z ^44^ | China | 4/22/2020 | All patients received chest CT | 72 | 46.2 | 54.2 | N/A | N/A | 2 | N/A | N/A | 15 | 8 |
| Lo^45^ | Macau | 3/15/2020 | N/A | 10 | 54 | 30 | N/A | N/A | 8 | N/A | N/A | 1 | 4 |
| Luo^46^ | China | 3/20/2020 | Patients with Only Gastrointestinal Symptoms | 183 | 53.8 | 56 | N/A | N/A | 68 | 180 | 183 | N/A | N/A |
| Mao^47^ | China | 4/10/2020 | N/A | 214 | 52.7 | 40.7 | N/A | N/A | 41 | N/A | N/A | N/A | 88 |
| Mi^48^ | China | 4/1/2020 | N/A | 10 | 68.4 | 20 | 1 | N/A | 0 | N/A | 0 | 4 | 7 |
| Mo^49^ | China | 3/16/2020 | Patients with Fractures | 155 | 54 | 55.5 | N/A | N/A | 7 | N/A | N/A | N/A | 37 |
| Nicoletti ^50^ | Italy | 5/15/2020 | Children | 42 | 6.21 | 47.6 | 13 | N/A | 2 | N/A | N/A | 8 | N/A |
| Nobel^51^ | USA | 4/12/2020 | N/A | 278 | N/A | 52 | 97 | N/A | 56 | N/A | N/A | N/A | N/A |
| Palaiodimos ^52^ | New York | 5/14/2020 | N/A | 200 | 64 | 49 | N/A | N/A | 66 | N/A | N/A | N/A | N/A |
| Pan^53^ | China | 4/14/2020 | N/A | 204 | 52.9 | 52 | 103 | 6 | 35 | 81 | 1 | N/A | N/A |
| Pung^54^ | Singapore | 3/16/2020 | N/A | 17 | 40 | 41 | N/A | N/A | 4 | N/A | N/A | N/A | 2 |
| Qian GQ^55^ | China | 3/17/2020 | N/A | 91 | 50 | 40.66 | N/A | N/A | 21 | 23 | N/A | N/A | 9 |
| Redd^56^ | USA | 4/22/2020 | N/A | 318 | 63.4 | 54.7 | 195 | N/A | 107 | 110 | N/A | N/A | N/A |
| Saeed^57^ | Norway | 4/28/2020 | Patients who present with Acute Abdomen | 9 | 48 | N/A | 9 | 9 | 1 | N/A | 4 | N/A | 0 |
| Shao^58^ | China | 4/6/2020 | Patients who Experienced Cardiac Arrest | 136 | 69 | 66.2 | N/A | N/A | 27 | N/A | N/A | N/A | 136 |
| Shen^59^ | China | 4/7/2020 | N/A | 9 | 8 | 33% | 2 | 2 | 2 | N/A | 1 | 2 | 0 |
| Shi S^60^ | China | 3/25/2020 | Patients with Cardiac Biomarker Lab Results | 416 | 64 | 49.3 | 16 | N/A | 16 | N/A | N/A | N/A | N/A |
| Shi^61^ | China | 2/24/2020 | Patients who had Serial Chest CT | 81 | 49.5 | 52 | N/A | N/A | 3 | 1 | N/A | 43 | N/A |
| Song^62^ | China | 2/6/2020 | Patients with COVID Pneumonia | 51 | 49 | 49 | N/A | N/A | 5 | 9 | N/A | N/A | 0 |
| Song^63^ | China | 4/24/2020 | N/A | 16 | 8.5 | 62.5 | 0 | 0 | 0 | 0 | 0 | N/A | 0 |
| Spiteri^64^ | Europe | 3/5/2020 | N/A | 38 | 42 | 65.6 | 1 | N/A | 1 | N/A | N/A | N/A | 4 |
| Sultan^65^ | USA | 4/25/2020 | Patients who receive ECMO | 10 | N/A | 70 | 3 | N/A | N/A | N/A | N/A | N/A | 10 |
| Sun Y^66^ | China | 3/25/2020 | N/A | 54 | 42 | 53.7 | 20 | N/A | N/A | N/A | N/A | N/A | N/A |
| Tan N ^67^ | China | 5/16/2020 | Patients with HTN treated with ACE/ARB | 100 | 67.4 | 51 | 24 | N/A | 12 | N/A | N/A | N/A | 87 |
| Tan^68^ | China | 4/10/2020 | N/A | 10 | 7 | 30 | 3 | N/A | N/A | N/A | 0 | 2 | 0 |
| Tang^69^ | China | 4/16/2020 | Patients who were Ultimately Discharged | 209 | N/A | 43.5 | 10 | N/A | 10 | N/A | N/A | N/A | N/A |
| Taxonera^70^ | Spain | 5/2/2020 | Patients with IBD | 12 | 52 | 25 | 9 | 2 | 9 | N/A | 2 | N/A | 8 |
| Toniati^71^ | Italy | 5/3/2020 | Patients with Severe ARDS | 100 | 62 | 88 | 9 | N/A | 9 | N/A | N/A | N/A | 100 |
| Tschopp ^72^ | Switzerland | 5/15/2020 | Transplant patients | 21 | 56 | 71 | 7 | N/A | 7 | N/A | N/A | N/A | N/A |
| Wan ^73^ | China | 4/15/2020 | N/A | 230 | 47.9 | 56 | 49 | 0 | 49 | N/A | 0 | N/A | 61 |
| Wang D^74^ | China | 2/7/2020 | Patients with COVID Pneumonia | 138 | 56 | 54.3 | N/A | 14 | 14 | 55 | 14 | N/A | 36 |
| Wang F^75^ | China | 5/1/2020 | Patients with Diabetes | 28 | 68.6 | 75 | N/A | N/A | 12 | 16 | N/A | N/A | 19 |
| Wang R ^76^ | China | 5/15/2020 | N/A | 125 | 38.76 | 56.8 | N/A | N/A | 50 | N/A | 3 | 27 | 25 |
| Wang X^77^ | China | 3/27/2020 | Non-Critically Ill Patients | 1012 | 50 | 51.8 | N/A | N/A | 152 | N/A | N/A | N/A | 0 |
| Wang X^78^ | China | 4/14/2020 | Medical Workers | 57 | 40 | 43.86 | 9 | N/A | 9 | N/A | N/A | N/A | 3 |
| Wang^79^ | China | 2/25/2020 | N/A | 18 | 39 | 55.6 | N/A | N/A | 3 | N/A | N/A | 4 | N/A |
| Wei X ^80^ | China | 5/17/2020 | N/A | 70 | 48.57 | 61.4 | 8 | N/A | 8 | N/A | N/A | 15 | N/A |
| Wei^81^ | China | 4/17/2020 | Health care workers with COVID Pneumonia | 84 | 37 | 33 | 53 | N/A | 26 | N/A | N/A | N/A | 0 |
| Wölfel^82^ | Germany | 4/1/2020 | N/A | 9 | N/A | N/A | 2 | N/A | 2 | N/A | 0 | N/A | 0 |
| Wu^83^ | China | 5/5/2020 | Pregnant Patients | 13 | 36 | 0 | 4 | N/A | 1 | N/A | 0 | 3 | 0 |
| Xia W^84^ | China | 3/5/2020 | N/A | 20 | 2.125 | 65 | N/A | N/A | 3 | N/A | N/A | 5 | N/A |
| Xia XY^85^ | China | 4/12/2020 | Familial cluster | 10 | 56.5 | 60 | 2 | 1 | 1 | N/A | 1 | N/A | 3 |
| Xie^86^ | China | 2/22/2020 | N/A | 9 | 38.3 | 44.4 | 1 | N/A | 1 | N/A | 0 | N/A | N/A |
| Xu T^87^ | China | 3/7/2020 | N/A | 51 | 42.37 | 49 | 5 | N/A | 5 | N/A | N/A | 4 | 0 |
| Xu^88^ | China | 2/19/2020 | N/A | 62 | 41 | 56 | 3 | N/A | 3 | N/A | N/A | 10 | 1 |
| Yang W^89^ | China | 2/26/2020 | N/A | 149 | 45.11 | 54.4 | N/A | N/A | 11 | N/A | N/A | 27 | 0 |
| Yang X^90^ | China | 2/21/2020 | ICU Patients with COVID Pneumonia | 52 | 59.7 | 67 | 2 | N/A | N/A | N/A | N/A | N/A | 52 |
| Ye^91^ | China | 4/18/2020 | Familial cluster | 5 | 40.4 | 60 | 1 | 0 | 1 | N/A | 0 | N/A | 1 |
| Yin^92^ | China | 4/30/2020 | N/A | 33 | 46 | 48 | 5 | N/A | 5 | N/A | N/A | 5 | 0 |
| Young^93^ | Singapore | 3/3/2020 | N/A | 18 | 47 | 50 | 3 | N/A | 3 | N/A | N/A | N/A | 2 |
| Yu^94^ | China | 3/24/2020 | Pregnant Women | 7 | 32 | 0 | 1 | N/A | 1 | N/A | 0 | 2 | 0 |
| Zhang G^95^ | China | 4/9/2020 | Diagnosed with COVID Pneumonia | 221 | 55 | 49 | N/A | N/A | 25 | 80 | N/A | N/A | 55 |
| Zhang H ^96^ | China | 5/8/2020 | N/A | 505 | 51.2 | 45.1 | 164 | N/A | 62 | 93 | N/A | N/A | 92 |
| Zhang J ^97^ | China | 4/15/2020 | N/A | 663 | 55.6 | 48.4 | N/A | N/A | 61 | N/A | N/A | 171 | 409 |
| Zhang Jin ^98^ | China | 4/21/2020 | Critically Ill ICU patients | 19 | 73 | 57.9 | 1 | N/A | 1 | N/A | N/A | N/A | 19 |
| Zhang JJ^99^ | China | 2/19/2020 | Patients with Respiratory Symptoms | 140 | 57 | 50.7 | 55 | N/A | 18 | 17 | N/A | N/A | 58 |
| Zhang R^100^ | China | 4/11/2020 | Patients with COVID Pneumonia | 120 | 45.4 | 36 | 10 | N/A | 7 | 3 | N/A | N/A | 30 |
| Zhang X ^101^ | China | 3/15/2020 | Patients who had a Chest Imaging | 645 | 34.9 | 45.8 | N/A | N/A | 53 | N/A | N/A | N/A | N/A |
| Zhang X^102^ | China | 3/20/2020 | Patients with CT Results Obtained | 645 | 45.2 | 50.9 | N/A | N/A | 53 | N/A | N/A | 81 | 64 |
| Zhao^103^ | China | 4/29/2020 | N/A | 91 | 46 | 53.8 | N/A | N/A | 14 | 11 | N/A | N/A | 30 |
| Zhou^104^ | China | 3/18/2020 | Patients with COVID Pneumonia | 254 | 50 | 45.3 | 66 | N/A | 46 | N/A | N/A | N/A | N/A |
| Zhou^105^ | China | 3/5/2020 | Patients with Respiratory Symptoms | 62 | 52.8 | 62.9 | 9 | N/A | N/A | N/A | N/A | N/A | N/A |
| Zhou^106^ | China | 3/9/2020 | Patients who were discharged or died | 191 | 56 | 62 | N/A | N/A | 9 | N/A | N/A | 59 | 119 |
| Zhu H ^107^ | China | 2/6/2020 | Pregnant mothers | 9 | 30 | 0 | 1 | N/A | 1 | N/A | 0 | N/A | N/A |
| Zhu Z ^108^ | China | 4/22/2020 | N/A | 127 | 50.9 | 35.43 | N/A | N/A | 43 | 59 | N/A | N/A | 16 |

All columns contain raw patient counts unless otherwise labeled. GI, gastrointestinal; COVID, coronavirus disease; LFTs, liver function tests (transaminases); CT, computed tomography; ECMO, extracorporeal membrane oxygenation; IBD, inflammatory bowel disease; ICU, intensive care unit; ARDS, acute respiratory distress syndrome; N/A, not applicable

**eTable 2.** Risk of bias ratings for included studies.

| Table of risk of bias (ROB) assessment: Each article was given a ROB rating (low=1, medium=2, high=3) according to the Newcastle Ottawa scale. Discrepancies were resolved by a third reviewer if necessary. | | | |
| --- | --- | --- | --- |
| Author | Reviewer 1 | Reviewer 2 | Reviewer 3 |
| Cai X^1^ | 3 | 3 | N/A |
| Chang^2^ | 3 | 3 | N/A |
| Chen G^3^ | 3 | 3 | N/A |
| Chen J^4^ | 3 | 3 | N/A |
| Chen J^5^ | 2 | 2 | N/A |
| Chen M^6^ | 3 | 3 | N/A |
| Chen N^7^ | 3 | 3 | N/A |
| Chen Q^8^ | 3 | 3 | N/A |
| Chen Q^9^ | 2 | 2 | N/A |
| Chen T^10^ | 3 | 3 | N/A |
| Chen Y^11^ | 3 | 3 | N/A |
| Cholankeril^12^ | 3 | 3 | N/A |
| Dai^13^ | 3 | 3 | N/A |
| Diao^14^ | 3 | 3 | N/A |
| Effenberger^15^ | 3 | 3 | N/A |
| Garazzino^16^ | 2 | 2 | N/A |
| Guan^17^ | 2 | 2 | N/A |
| Hajifathalian^18^ | 2 | 2 | N/A |
| Han C^19^ | 3 | 3 | N/A |
| Han R^20^ | 3 | 3 | N/A |
| Han YN^21^ | 3 | 3 | N/A |
| He^22^ | 2 | 2 | N/A |
| Hossain^23^ | 3 | 3 | N/A |
| Huang C^24^ | 3 | 3 | N/A |
| Huang L^25^ | 3 | 3 | N/A |
| Huang LE^26^ | 2 | 2 | N/A |
| Huang Y^27^ | 3 | 3 | N/A |
| Jin^28^ | 3 | 3 | N/A |
| Kim^29^ | 3 | 3 | N/A |
| Klopfenstein^30^ | 3 | 3 | N/A |
| Lechien^31^ | 2 | 2 | N/A |
| Lei P^32^ | 3 | 3 | N/A |
| Lei Z^33^ | 3 | 3 | N/A |
| Li^34^ | 3 | 3 | N/A |
| Li^35^ | 3 | 3 | N/A |
| Lian^36^ | 2 | 2 | N/A |
| Lin^37^ | 3 | 3 | N/A |
| Liu F^38^ | 3 | 3 | N/A |
| Liu J^39^ | 3 | 3 | N/A |
| Liu J-Y^40^ | 2 | 2 | N/A |
| Liu K^41^ | 3 | 3 | N/A |
| Liu W^42^ | 3 | 3 | N/A |
| Liu Y^43^ | 3 | 3 | N/A |
| Lo^45^ | 3 | 3 | N/A |
| Luo^46^ | 3 | 3 | N/A |
| Mao^47^ | 3 | 3 | N/A |
| Mi^48^ | 3 | 3 | N/A |
| Mo^49^ | 3 | 3 | N/A |
| Nicoletti^50^ | 3 | 3 | N/A |
| Nobel^51^ | 2 | 2 | N/A |
| Palaiodimos^52^ | 2 | 2 | N/A |
| Pan^53^ | 2 | 2 | N/A |
| Pung^54^ | 3 | 3 | N/A |
| Qian GQ^55^ | 3 | 3 | N/A |
| Redd^56^ | 2 | 2 | N/A |
| Saeed^57^ | 3 | 3 | N/A |
| Shao^58^ | 2 | 3 | 2 |
| Shen^59^ | 3 | 3 | N/A |
| Shi S^60^ | 2 | 2 | N/A |
| Shi^61^ | 3 | 3 | N/A |
| Song^62^ | 3 | 3 | N/A |
| Song^63^ | 3 | 3 | N/A |
| Spiteri^64^ | 3 | 3 | N/A |
| Sultan^65^ | 3 | 3 | N/A |
| Sun Y^66^ | 2 | 2 | N/A |
| Tan N^67^ | 3 | 3 | N/A |
| Tan^68^ | 3 | 3 | N/A |
| Tang^69^ | 3 | 3 | N/A |
| Taxonera^70^ | 3 | 3 | N/A |
| Toniati^71^ | 3 | 3 | N/A |
| Tschopp^72^ | 3 | 3 | N/A |
| Wan^73^ | 2 | 2 | N/A |
| Wang D^74^ | 3 | 3 | N/A |
| Wang F^75^ | 3 | 3 | N/A |
| Wang R^76^ | 2 | 2 | 3 |
| Wang X^77^ | 2 | 3 | 2 |
| Wang X^78^ | 3 | 3 | N/A |
| Wang^79^ | 3 | 3 | N/A |
| Wei X^80^ | 3 | 3 | N/A |
| Wei^81^ | 3 | 3 | N/A |
| Wölfel^82^ | 3 | 2 | 2 |
| Wu^83^ | 3 | 3 | N/A |
| Xia W^84^ | 3 | 3 | N/A |
| Xia XY^85^ | 3 | 3 | N/A |
| Xie^86^ | 3 | 3 | N/A |
| Xu T^87^ | 3 | 2 | 3 |
| Xu^88^ | 3 | 3 | N/A |
| Yang W^89^ | 2 | 2 | N/A |
| Yang X^90^ | 3 | 3 | N/A |
| Ye^91^ | 3 | 3 | N/A |
| Yin^92^ | 3 | 3 | N/A |
| Young^93^ | 3 | 3 | N/A |
| Yu^94^ | 3 | 3 | N/A |
| Zhang G^95^ | 2 | 3 | 2 |
| Zhang H^96^ | 3 | 3 | N/A |
| Zhang J^97^ | 2 | 2 | N/A |
| Zhang Jin^98^ | 3 | 3 | N/A |
| Zhang JJ^99^ | 3 | 3 | N/A |
| Zhang R^100^ | 2 | 3 | 2 |
| Zhang X ^101^ | 3 | 3 | N/A |
| Zhang X^102^ | 3 | 3 | N/A |
| Zhao^103^ | 3 | 3 | N/A |
| Zhou^104^ | 2 | 2 | N/A |
| Zhou^105^ | 3 | 3 | N/A |
| Zhou^106^ | 2 | 2 | N/A |
| Zhu H^107^ | 3 | 3 | N/A |
| Zhu Z^108^ | 3 | 3 | N/A |

**eFigure 1**. Proportion of COVID-19 patients with any GI symptom.

**Legend:** Overall prevalence of GI symptoms in patients with COVID-19 was 20% (95%CI 15-24%). 17% (95%CI 13-21%) endorsed GI symptoms if anorexia was not included in the definition of GI symptoms, versus 32% (95%CI 22-43%) if anorexia was part of the definition (p=0.01 for difference).

GI, gastrointestinal; COVID, coronavirus disease; CI, confidence interval

**eFigure 2.** Proportion of COVID-19 patients with GI symptoms as part of initial presentation.

**Legend:** Overall prevalence of GI symptoms at presentation in patients with COVID-19 was 19% (95%CI 14-25%). 17% (95%CI 12-22%) endorsed GI symptoms if anorexia was not included in the definition of GI symptoms, versus 24% (95%CI 15-34%) if anorexia was part of the definition (p=0.29 for difference).

GI, gastrointestinal; COVID, coronavirus disease; CI, confidence interval

**eFigure 3.** Proportion of COVID-19 patients with only GI symptoms, including patients with or without fever.

**Legend:** Overall prevalence of COVID-19 infection with a period of only GI symptoms, with or without fever but where the presence of fever was defined, was 2% (95%CI 0-6%). Whether anorexia was included in the definition of GI symptoms or not did not affect the proportion with GI symptoms, p=0.73.

GI, gastrointestinal; COVID, coronavirus disease; CI, confidence interval

**eFigure 4.** Proportion of COVID-19 patients with only GI symptoms, regardless of definition of fever.

**Legend:** Overall prevalence of COVID-19 infection with a period of only GI symptoms, with or without fever but including studies where the presence of fever was undefined, was 2% (95%CI 0-6%). Whether anorexia was included in the definition of GI symptoms or not did not affect the proportion with GI symptoms, p=0.91. GI, gastrointestinal; COVID, coronavirus disease; CI, confidence interval

**eFigure 5**. Funnel plot of proportion of COVID-19 patients with only GI symptoms (by standard error of proportion).

**Legend.** Plotting the double arcsine transformed proportion of COVID-19 patients with only GI symptoms (with or without fever, x-axis) versus the standard error of that transformed proportion (y-axis), there is no funnel plot asymmetry (p=0.12), indicating there is no evidence of small study effects such as publication bias for this outcome. This was also true when using sample size alone as the y-axis.

GI, gastrointestinal; COVID, coronavirus disease

**eFigure 6.** Proportion of all COVID-19 patients with diarrhea, by whether cohort restricted to respiratory symptoms or not.

**Legend:** Overall prevalence of diarrhea in COVID-19 infection was 13% (95%CI 11-16%). This was only 9% (95%CI 7-12%) in studies restricted to patients with respiratory symptoms, but 15% (95%CI 12-18%) in studies not restricted to patients with respiratory symptoms (p<0.01 for difference).

COVID, coronavirus disease; CI, confidence interval

**eFigure 7.** Proportion of all COVID-19 patients with nausea and/or vomiting.

**Legend:** Overall prevalence of nausea and/or vomiting in COVID-19 infection was 10% (95%CI 7-12%).

COVID, coronavirus disease; CI, confidence interval

**eFigure 8.** Proportion of all COVID-19 patients with vomiting.

**Legend:** Overall prevalence of vomiting in COVID-19 infection was 4% (95%CI 3-6%).

COVID, coronavirus disease; CI, confidence interval

**eFigure 9**. Proportion of all COVID-19 patients with abdominal pain.

**Legend:** Overall prevalence of abdominal pain in COVID-19 infection was 4% (95%CI 2-6%). This was not significantly different in studies restricted to patients with respiratory symptoms or not (p=0.13 for difference).

COVID, coronavirus disease; CI, confidence interval

**eFigure 10.** Proportion of all COVID-19 patients with anorexia / loss of appetite.

**Legend:** Overall prevalence of anorexia in COVID-19 infection was 21% (95%CI 15-27%). This was only 10% (95%CI 2-23%) in studies restricted to patients with respiratory symptoms, but 25% (95%CI 18-32%) in studies not restricted to patients with respiratory symptoms (p=0.05 for difference).

COVID, coronavirus disease; CI, confidence interval

**eFigure 11.** Proportion of all COVID-19 patients with diarrhea, by continent.

**Legend:** Prevalence of diarrhea in COVID-19 infection was only 11% (95%CI 9-14%) in studies from Asia, but 24% (95%CI 18-31%) in non-Asian studies (p<0.01 for difference).

COVID, coronavirus disease; CI, confidence interval

**eFigure 12**. Proportion of all COVID-19 patients with nausea or vomiting, by continent.

**Legend:** Prevalence of nausea or vomiting in COVID-19 infection was only 8% (95%CI 6-10%) in studies from Asia, but 18% (95%CI 15-22%) in non-Asian studies (p<0.01 for difference).

COVID, coronavirus disease; CI, confidence interval

**eFigure 13.** Proportion of all COVID patients with abdominal pain, by continent.

**Legend:** Prevalence of abdominal pain in COVID-19 infection was only 2% (95%CI 1-3%) in studies from Asia, but 12% (95%CI 6-20%) in non-Asian studies (p<0.01 for difference).

COVID, coronavirus disease; CI, confidence interval

**eFigure 14.** Proportion of all COVID-19 patients with any GI symptoms, by continent.

**Legend:** Prevalence of any GI symptoms in COVID-19 infection was only 17% (95%CI 13-22%) in studies from Asia, but 31% (95%CI 20-43%) in non-Asian studies (p=0.03 for difference).

GI, gastrointestinal; COVID, coronavirus disease; CI, confidence interval

**eFigure 15.** Proportion of all COVID-19 patients with anorexia, by continent.

**Legend:** Prevalence of anorexia in COVID-19 infection was only 19% (95%CI 13-26%) in studies from Asia, but 30% (95%CI 18-45%) in non-Asian studies (p=0.14 for difference).

GI, gastrointestinal; COVID, coronavirus disease; CI, confidence interval

**eFigure 16.** Proportion of all COVID-19 patients with diarrhea, by publication date.

**Legend:** Prevalence of diarrhea in COVID-19 infection was only 9% (95%CI 7-12%) in studies published on or before April 1, but 16% (95%CI 13-20%) in studies published after April 1 (p<0.01 for difference).

COVID, coronavirus disease; CI, confidence interval

**Figure 17.** Proportion of all COVID-19 patients with nausea or vomiting, by publication date.

**Legend:** Prevalence of nausea or vomiting in COVID-19 infection was only 6% (95%CI 4-9%) in studies published on or before April 1, but 12% (95%CI 8-16%) in studies published after April 11 (p=0.01 for difference).

COVID, coronavirus disease; CI, confidence interval

**eFigure 18.** Proportion of patients with any GI symptoms, by publication date.

**Legend:** Prevalence of any GI symptoms in COVID-19 infection was only 13% (95%CI 9-19%) in studies published on or before April 1, but 24% (95%CI 18-34%) in studies published after April 1 (p=0.01 for difference).

GI, gastrointestinal; COVID, coronavirus disease; CI, confidence interval

**eFigure 19.** Proportion of all COVID-19 patients with abdominal pain, by publication date.

**Legend:** Prevalence of abdominal pain in COVID-19 infection was 3% (95%CI 2-5%) in studies published on or before April 1, and 4% (95%CI 2-7%) in studies published after April 1 (p=0.38 for difference).

COVID, coronavirus disease; CI, confidence interval

**eFigure 20.** Proportion of all COVID-19 patients with anorexia, by publication date.

**Legend:** Prevalence of anorexia in COVID-19 infection was only 15% (95%CI 8-24%) in studies published on or before April 1, but 24% (95%CI 16-32%) in studies published after April 1 (p=0.14 for difference).

COVID, coronavirus disease; CI, confidence interval

**eFigure 21.** Proportion of all COVID-19 patients with elevated transaminases, by continent.

**Legend:** Prevalence of elevated transaminases in COVID-19 infection was only 22% (95%CI 17-27%) in studies from East Asia, but 61% (95%CI 58-64%) in North American studies (p<0.01 for this comparison), though there were only two North American studies. When incorporating the single European study, the non-Asian pooled prevalence was 41% (95%CI 17-67%, p=0.16 for difference from Asian studies).

COVID, coronavirus disease; CI, confidence interval

**eFigure 22.** Proportion of all COVID-19 patients with elevated transaminases, by publication date.

**Legend:** Prevalence of any GI symptoms in COVID-19 infection was 27% (95%CI 20-34%) in studies published on or before April 15, and 21% (95%CI 12-32%) in studies published after April 1, a non-significant difference (p=0.39) in the opposite direction to the digestive symptoms.

COVID, coronavirus disease; CI, confidence interval

**eFigure 23**. Odds of severe COVID-19 in patients with GI symptoms compared to those without.

**Legend:** Odds of severe COVID were significantly greater (OR 2.07, 95%CI 1.34-3.18) in patients with GI symptoms compared to those without.

GI, gastrointestinal; COVID, coronavirus disease; OR, odds ratio; CI, confidence interval

**eFigure 24.** Odds of severe COVID-19 in patients with diarrhea.

**Legend:** Odds of severe COVID were greater (OR 1.60, 95%CI 0.90-2.82) in patients with diarrhea compared to those without, but this was not statistically significant.

COVID, coronavirus disease; OR, odds ratio; CI, confidence interval

**eFigure 25.** Odds of severe COVID-19 in patients with nausea or vomiting.

**Legend:** Odds of severe COVID were not statistically significantly greater (OR 1.20, 95%CI 0.56-2.55) in patients with nausea or vomiting compared to those without.

COVID, coronavirus disease; OR, odds ratio; CI, confidence interval

**eFigure 26.** Odds of severe COVID-19 in patients with abdominal pain.

**Legend:** Odds of severe COVID were not statistically significantly greater (OR 1.54, 95%CI 0.65-3.67) in patients with abdominal pain compared to those without.

COVID, coronavirus disease; OR, odds ratio; CI, confidence interval

**REFERENCES**

1. Cai X, Ma Y, Li S, Chen Y, Rong Z, Li W. Clinical Characteristics of 5 COVID-19 Cases With Non-respiratory Symptoms as the First Manifestation in Children. *Frontiers in Pediatrics.* 2020;8.

2. Chang D, Lin M, Wei L, et al. Epidemiologic and Clinical Characteristics of Novel Coronavirus Infections Involving 13 Patients Outside Wuhan, China. *Jama.* 2020;323(11):1092-1093.

3. Chen H, Guo J, Wang C, et al. Clinical characteristics and intrauterine vertical transmission potential of COVID-19 infection in nine pregnant women: a retrospective review of medical records. *The Lancet.* 2020;395(10226):809-815.

4. Chen J, Zhang ZZ, Chen YK, et al. The clinical and immunological features of pediatric COVID-19 patients in China. *Genes and Diseases.* 2020.

5. Chen J, Qi T, Liu L, et al. Clinical progression of patients with COVID-19 in Shanghai, China. *J Infect.* 2020;80(5):e1-e6.

6. Chen M, An W, Xia F, et al. Clinical Characteristics of Re-hospitalized Patients with COVID-19 in China. *J Med Virol.* 2020.

7. Chen N, Zhou M, Dong X, et al. Epidemiological and clinical characteristics of 99 cases of 2019 novel coronavirus pneumonia in Wuhan, China: a descriptive study. *Lancet.* 2020;395(10223):507-513.

8. Chen Q, Quan B, Li X, et al. A report of clinical diagnosis and treatment of nine cases of coronavirus disease 2019. *J Med Virol.* 2020.

9. Chen Q, Zheng Z, Zhang C, et al. Clinical characteristics of 145 patients with corona virus disease 2019 (COVID-19) in Taizhou, Zhejiang, China. *Infection.* 2020.

10. Chen T, Wu D, Chen H, et al. Clinical characteristics of 113 deceased patients with coronavirus disease 2019: retrospective study. *Bmj.* 2020;368:m1091.

11. Chen Y, Chen L, Deng Q, et al. The Presence of SARS-CoV-2 RNA in Feces of COVID-19 Patients. *J Med Virol.* 2020.

12. Cholankeril G, Podboy A, Aivaliotis VI, et al. High Prevalence of Concurrent Gastrointestinal Manifestations in Patients with SARS-CoV-2: Early Experience from California. *Gastroenterology.* 2020.

13. Dai H, Zhang X, Xia J, et al. High-resolution Chest CT Features and Clinical Characteristics of Patients Infected with COVID-19 in Jiangsu, China. *International Journal of Infectious Diseases.* 2020;95:106-112.

14. Diao K, Han P, Pang T, Li Y, Yang Z. HRCT imaging features in representative imported cases of 2019 novel coronavirus pneumonia. *Precision Clinical Medicine.* 2020;3(1):9-13.

15. Effenberger M, Grabherr F, Mayr L, et al. Faecal calprotectin indicates intestinal inflammation in COVID-19. *Gut.* 2020.

16. Garazzino S, Montagnani C, Dona D, et al. Multicentre Italian study of SARS-CoV-2 infection in children and adolescents, preliminary data as at 10 April 2020. *Euro Surveill.* 2020;25(18).

17. Guan WJ, Ni ZY, Hu Y, et al. Clinical Characteristics of Coronavirus Disease 2019 in China. *N Engl J Med.* 2020.

18. Hajifathalian K, Krisko T, Mehta A, et al. Gastrointestinal and Hepatic Manifestations of 2019 Novel Coronavirus Disease in a Large Cohort of Infected Patients From New York: Clinical Implications. *Gastroenterology.* 2020.

19. Han C, Duan C, Zhang S, et al. Digestive Symptoms in COVID-19 Patients With Mild Disease Severity: Clinical Presentation, Stool Viral RNA Testing, and Outcomes. *Am J Gastroenterol.* 2020.

20. Han R, Huang L, Jiang H, Dong J, Peng H, Zhang D. Early Clinical and CT Manifestations of Coronavirus Disease 2019 (COVID-19) Pneumonia. *AJR Am J Roentgenol.* 2020:1-6.

21. Han YN, Feng ZW, Sun LN, et al. A comparative-descriptive analysis of clinical characteristics in 2019-coronavirus-infected children and adults. *J Med Virol.* 2020.

22. He R, Lu Z, Zhang L, et al. The clinical course and its correlated immune status in COVID-19 pneumonia. *Journal of Clinical Virology.* 2020;127.

23. Hossain R, Lazarus MS, Roudenko A, et al. CT Scans Obtained for Nonpulmonary Indications: Associated Respiratory Findings of COVID-19. *Radiology.* 2020:201743.

24. Huang C, Wang Y, Li X, et al. Clinical features of patients infected with 2019 novel coronavirus in Wuhan, China. *The Lancet.* 2020;395(10223):497-506.

25. Huang L, Jiang J, Li X, Zhou Y, Xu M, Zhou J. Initial CT imaging characters of an imported family cluster of COVID-19. *Clinical Imaging.* 2020;65:78-81.

26. Huang L, Zhang X, Zhang X, et al. Rapid asymptomatic transmission of COVID-19 during the incubation period demonstrating strong infectivity in a cluster of youngsters aged 16-23 years outside Wuhan and characteristics of young patients with COVID-19: A prospective contact-tracing study. *Journal of Infection.* 2020;80(6):e1-e13.

27. Huang Y, Tu M, Wang S, et al. Clinical characteristics of laboratory confirmed positive cases of SARS-CoV-2 infection in Wuhan, China: A retrospective single center analysis. *Travel Med Infect Dis.* 2020:101606.

28. Jin X, Lian JS, Hu JH, et al. Epidemiological, clinical and virological characteristics of 74 cases of coronavirus-infected disease 2019 (COVID-19) with gastrointestinal symptoms. *Gut.* 2020.

29. Kim ES, Chin BS, Kang CK, et al. Clinical Course and Outcomes of Patients with Severe Acute Respiratory Syndrome Coronavirus 2 Infection: a Preliminary Report of the First 28 Patients from the Korean Cohort Study on COVID-19. *J Korean Med Sci.* 2020;35(13):e142.

30. Klopfenstein T, Kadiane-Oussou NJ, Royer PY, Toko L, Gendrin V, Zayet S. Diarrhea: An underestimated symptom in Coronavirus disease 2019. *Clin Res Hepatol Gastroenterol.* 2020.

31. Lechien JR, Chiesa-Estomba CM, Place S, et al. Clinical and Epidemiological Characteristics of 1,420 European Patients with mild-to-moderate Coronavirus Disease 2019. *J Intern Med.* 2020.

32. Lei P, Huang Z, Liu G, et al. Clinical and computed tomographic (CT) images characteristics in the patients with COVID-19 infection: What should radiologists need to know? *J Xray Sci Technol.* 2020.

33. Lei Z, Cao H, Jie Y, et al. A cross-sectional comparison of epidemiological and clinical features of patients with coronavirus disease (COVID-19) in Wuhan and outside Wuhan, China. *Travel Med Infect Dis.* 2020:101664.

34. Li J, Wang X, Chen J, Zuo X, Zhang H, Deng A. COVID-19 infection may cause ketosis and ketoacidosis. *Diabetes Obes Metab.* 2020.

35. Li K, Wu J, Wu F, et al. The Clinical and Chest CT Features Associated With Severe and Critical COVID-19 Pneumonia. *Invest Radiol.* 2020;55(6):327-331.

36. Lian J, Jin X, Hao S, et al. Epidemiological, clinical, and virological characteristics of 465 hospitalized cases of coronavirus disease 2019 (COVID-19) from Zhejiang province in China. *Influenza Other Respir Viruses.* 2020.

37. Lin L, Jiang X, Zhang Z, et al. Gastrointestinal symptoms of 95 cases with SARS-CoV-2 infection. *Gut.* 2020.

38. Liu F, Xu A, Zhang Y, et al. Patients of COVID-19 may benefit from sustained Lopinavir-combined regimen and the increase of Eosinophil may predict the outcome of COVID-19 progression. *International Journal of Infectious Diseases.* 2020;95:183-191.

39. Liu J, Li S, Liu J, et al. Longitudinal characteristics of lymphocyte responses and cytokine profiles in the peripheral blood of SARS-CoV-2 infected patients. *EBioMedicine.* 2020;55.

40. Liu JY, Chen TJ, Hwang SJ. Analysis of imported cases of covid-19 in taiwan: A nationwide study. *International Journal of Environmental Research and Public Health.* 2020;17(9).

41. Liu K, Fang YY, Deng Y, et al. Clinical characteristics of novel coronavirus cases in tertiary hospitals in Hubei Province. *Chin Med J (Engl).* 2020.

42. Liu W, Zhang Q, Chen J, et al. Detection of Covid-19 in children in early january 2020 in Wuhan, China. *New England Journal of Medicine.* 2020;382(14):1370-1372.

43. Liu Y, Yang Y, Zhang C, et al. Clinical and biochemical indexes from 2019-nCoV infected patients linked to viral loads and lung injury. *Sci China Life Sci.* 2020;63(3):364-374.

44. Liu Z, Jin C, Wu CC, et al. Association between initial chest CT or clinical features and clinical course in patients with coronavirus disease 2019 Pneumonia. *Korean Journal of Radiology.* 2020;21(6):736-745.

45. Lo IL, Lio CF, Cheong HH, et al. Evaluation of SARS-CoV-2 RNA shedding in clinical specimens and clinical characteristics of 10 patients with COVID-19 in Macau. *Int J Biol Sci.* 2020;16(10):1698-1707.

46. Luo S, Zhang X, Xu H. Don't Overlook Digestive Symptoms in Patients With 2019 Novel Coronavirus Disease (COVID-19). *Clin Gastroenterol Hepatol.* 2020;18(7):1636-1637.

47. Mao L, Jin H, Wang M, et al. Neurologic Manifestations of Hospitalized Patients With Coronavirus Disease 2019 in Wuhan, China. *JAMA Neurol.* 2020.

48. Mi B, Chen L, Xiong Y, Xue H, Zhou W, Liu G. Characteristics and Early Prognosis of COVID-19 Infection in Fracture Patients. *J Bone Joint Surg Am.* 2020.

49. Mo P, Xing Y, Xiao Y, et al. Clinical characteristics of refractory COVID-19 pneumonia in Wuhan, China. *Clin Infect Dis.* 2020.

50. Nicoletti A, Talarico V, Sabetta L, et al. Screening of COVID-19 in children admitted to the hospital for acute problems: Preliminary data. *Acta Biomedica.* 2020;91(2):75-79.

51. Nobel YR, Phipps M, Zucker J, et al. Gastrointestinal Symptoms and COVID-19: Case-Control Study from the United States. *Gastroenterology.* 2020.

52. Palaiodimos L, Kokkinidis DG, Li W, et al. Severe obesity is associated with higher in-hospital mortality in a cohort of patients with COVID-19 in the Bronx, New York. *Metabolism: Clinical and Experimental.* 2020;108.

53. Pan L, Mu M, Yang P, et al. Clinical Characteristics of COVID-19 Patients With Digestive Symptoms in Hubei, China: A Descriptive, Cross-Sectional, Multicenter Study. *Am J Gastroenterol.* 2020.

54. Pung R, Chiew CJ, Young BE, et al. Investigation of three clusters of COVID-19 in Singapore: implications for surveillance and response measures. *The Lancet.* 2020;395(10229):1039-1046.

55. Qian GQ, Yang NB, Ding F, et al. Epidemiologic and Clinical Characteristics of 91 Hospitalized Patients with COVID-19 in Zhejiang, China: A retrospective, multi-centre case series. *Qjm.* 2020.

56. Redd WD, Zhou JC, Hathorn KE, et al. Prevalence and Characteristics of Gastrointestinal Symptoms in Patients with SARS-CoV-2 Infection in the United States: A Multicenter Cohort Study. *Gastroenterology.* 2020.

57. Saeed U, Sellevoll HB, Young VS, Sandbaek G, Glomsaker T, Mala T. Covid-19 may present with acute abdominal pain. *Br J Surg.* 2020.

58. Shao F, Xu S, Ma X, et al. In-hospital cardiac arrest outcomes among patients with COVID-19 pneumonia in Wuhan, China. *Resuscitation.* 2020;151:18-23.

59. Shen Q, Guo W, Guo T, et al. Novel coronavirus infection in children outside of Wuhan, China. *Pediatr Pulmonol.* 2020.

60. Shi S, Qin M, Shen B, et al. Association of Cardiac Injury With Mortality in Hospitalized Patients With COVID-19 in Wuhan, China. *JAMA Cardiol.* 2020.

61. Shi H, Han X, Jiang N, et al. Radiological findings from 81 patients with COVID-19 pneumonia in Wuhan, China: a descriptive study. *Lancet Infect Dis.* 2020;20(4):425-434.

62. Song F, Shi N, Shan F, et al. Emerging 2019 novel coronavirus (2019-NCoV) pneumonia. *Radiology.* 2020;295(1):210-217.

63. Song W, Li J, Zou N, Guan W, Pan J, Xu W. Clinical features of pediatric patients with coronavirus disease (COVID-19). *Journal of Clinical Virology.* 2020;127.

64. Spiteri G, Fielding J, Diercke M, et al. First cases of coronavirus disease 2019 (COVID-19) in the WHO European Region, 24 January to 21 February 2020. *Euro Surveill.* 2020;25(9).

65. Sultan I, Habertheuer A, Usman AA, et al. The role of extracorporeal life support for patients with COVID-19: Preliminary results from a statewide experience. *J Card Surg.* 2020.

66. Sun Y, Koh V, Marimuthu K, et al. Epidemiological and Clinical Predictors of COVID-19. *Clin Infect Dis.* 2020.

67. Tan ND, Qiu Y, Xing XB, Ghosh S, Chen MH, Mao R. Associations between Angiotensin Converting Enzyme Inhibitors and Angiotensin II Receptor Blocker Use, Gastrointestinal Symptoms, and Mortality among Patients with COVID-19. *Gastroenterology.* 2020.

68. Tan YP, Tan BY, Pan J, Wu J, Zeng SZ, Wei HY. Epidemiologic and clinical characteristics of 10 children with coronavirus disease 2019 in Changsha, China. *J Clin Virol.* 2020;127:104353.

69. Tang X, Zhao S, He D, et al. Positive RT-PCR tests among discharged COVID-19 patients in Shenzhen, China. *Infect Control Hosp Epidemiol.* 2020:1-7.

70. Taxonera C, Sagastagoitia I, Alba C, Manas N, Olivares D, Rey E. 2019 Novel Coronavirus Disease (COVID-19) in patients with Inflammatory Bowel Diseases. *Aliment Pharmacol Ther.* 2020.

71. Toniati P, Piva S, Cattalini M, et al. Tocilizumab for the treatment of severe COVID-19 pneumonia with hyperinflammatory syndrome and acute respiratory failure: A single center study of 100 patients in Brescia, Italy. *Autoimmun Rev.* 2020:102568.

72. Tschopp J, L'Huillier AG, Mombelli M, et al. First experience of SARS-CoV-2 infections in solid organ transplant recipients in the Swiss Transplant Cohort Study. *Am J Transplant.* 2020.

73. Wan Y, Li J, Shen L, et al. Enteric involvement in hospitalised patients with COVID-19 outside Wuhan. *The Lancet Gastroenterology and Hepatology.* 2020;5(6):534-535.

74. Wang D, Hu B, Hu C, et al. Clinical Characteristics of 138 Hospitalized Patients With 2019 Novel Coronavirus-Infected Pneumonia in Wuhan, China. *Jama.* 2020.

75. Wang F, Yang Y, Dong K, et al. CLINICAL CHARACTERISTICS OF 28 PATIENTS WITH DIABETES AND COVID-19 IN WUHAN, CHINA. *Endocr Pract.* 2020.

76. Wang R, Pan M, Zhang X, et al. Epidemiological and clinical features of 125 Hospitalized Patients with COVID-19 in Fuyang, Anhui, China. *International Journal of Infectious Diseases.* 2020;95:421-428.

77. Wang X, Fang J, Zhu Y, et al. Clinical characteristics of non-critically ill patients with novel coronavirus infection (COVID-19) in a Fangcang Hospital. *Clinical Microbiology and Infection.* 2020.

78. Wang X, Liu W, Zhao J, et al. Clinical characteristics of 80 hospitalized frontline medical workers infected with COVID-19 in Wuhan, China. *J Hosp Infect.* 2020.

79. Wang L, Gao YH, Lou LL, Zhang GJ. The clinical dynamics of 18 cases of COVID-19 outside of Wuhan, China. *Eur Respir J.* 2020;55(4).

80. Wei XY, Jing D, Jia B, et al. Characteristics of in peripheral blood of 70 hospitalized patients and 8 diarrhea patients with COVID-19. *Int J Med Sci.* 2020;17(9):1142-1146.

81. Wei XS, Wang X, Niu YR, et al. Diarrhea is associated with prolonged symptoms and viral carriage in COVID-19. *Clin Gastroenterol Hepatol.* 2020.

82. Wölfel R, Corman VM, Guggemos W, et al. Virological assessment of hospitalized patients with COVID-2019. *Nature.* 2020;581(7809):465-469.

83. Wu Y, Liu C, Dong L, et al. Coronavirus disease 2019 among pregnant Chinese women: Case series data on the safety of vaginal birth and breastfeeding. *BJOG : an international journal of obstetrics and gynaecology.* 2020.

84. Xia W, Shao J, Guo Y, Peng X, Li Z, Hu D. Clinical and CT features in pediatric patients with COVID-19 infection: Different points from adults. *Pediatric Pulmonology.* 2020;55(5):1169-1174.

85. Xia XY, Wu J, Liu HL, Xia H, Jia B, Huang WX. Epidemiological and initial clinical characteristics of patients with family aggregation of COVID-19. *Journal of Clinical Virology.* 2020;127.

86. Xie C, Jiang L, Huang G, et al. Comparison of different samples for 2019 novel coronavirus detection by nucleic acid amplification tests. *International Journal of Infectious Diseases.* 2020;93:264-267.

87. Xu T, Chen C, Zhu Z, et al. Clinical features and dynamics of viral load in imported and non-imported patients with COVID-19. *International Journal of Infectious Diseases.* 2020;94:68-71.

88. Xu XW, Wu XX, Jiang XG, et al. Clinical findings in a group of patients infected with the 2019 novel coronavirus (SARS-Cov-2) outside of Wuhan, China: Retrospective case series. *The BMJ.* 2020;368.

89. Yang W, Cao Q, Qin L, et al. Clinical characteristics and imaging manifestations of the 2019 novel coronavirus disease (COVID-19):A multi-center study in Wenzhou city, Zhejiang, China. *J Infect.* 2020;80(4):388-393.

90. Yang X, Yu Y, Xu J, et al. Clinical course and outcomes of critically ill patients with SARS-CoV-2 pneumonia in Wuhan, China: a single-centered, retrospective, observational study. *Lancet Respir Med.* 2020;8(5):475-481.

91. Ye F, Xu S, Rong Z, et al. Delivery of infection from asymptomatic carriers of COVID-19 in a familial cluster. *International journal of infectious diseases : IJID : official publication of the International Society for Infectious Diseases.* 2020.

92. Yin S, Peng Y, Ren Y, et al. The implications of preliminary screening and diagnosis: Clinical characteristics of 33 mild patients with SARS-CoV-2 infection in Hunan, China. *J Clin Virol.* 2020;128:104397.

93. Young BE, Ong SWX, Kalimuddin S, et al. Epidemiologic Features and Clinical Course of Patients Infected With SARS-CoV-2 in Singapore. *Jama.* 2020.

94. Yu N, Li W, Kang Q, et al. Clinical features and obstetric and neonatal outcomes of pregnant patients with COVID-19 in Wuhan, China: a retrospective, single-centre, descriptive study. *The Lancet Infectious Diseases.* 2020.

95. Zhang G, Hu C, Luo L, et al. Clinical features and short-term outcomes of 221 patients with COVID-19 in Wuhan, China. *J Clin Virol.* 2020;127:104364.

96. Zhang H, Liao YS, Gong J, Liu J, Xia X, Zhang H. Clinical characteristics of coronavirus disease (COVID-19) patients with gastrointestinal symptoms: A report of 164 cases. *Digestive and Liver Disease.* 2020.

97. Zhang J, Wang X, Jia X, et al. Risk factors for disease severity, unimprovement, and mortality in COVID-19 patients in Wuhan, China. *Clinical Microbiology and Infection.* 2020;26(6):767-772.

98. Zhang J, Liu P, Wang M, et al. The clinical data from 19 critically ill patients with coronavirus disease 2019: a single-centered, retrospective, observational study. *Z Gesundh Wiss.* 2020:1-4.

99. Zhang JJ, Dong X, Cao YY, et al. Clinical characteristics of 140 patients infected with SARS-CoV-2 in Wuhan, China. *Allergy.* 2020.

100. Zhang R, Ouyang H, Fu L, et al. CT features of SARS-CoV-2 pneumonia according to clinical presentation: a retrospective analysis of 120 consecutive patients from Wuhan city. *Eur Radiol.* 2020.

101. Zhang X, Cai H, Hu J, et al. Epidemiological, clinical characteristics of cases of SARS-CoV-2 infection with abnormal imaging findings. *International Journal of Infectious Diseases.* 2020.

102. Zhang X, Cai H, Hu J, et al. Epidemiological, clinical characteristics of cases of SARS-CoV-2 infection with abnormal imaging findings. *Int J Infect Dis.* 2020;94:81-87.

103. Zhao XY, Xu XX, Yin HS, et al. Clinical characteristics of patients with 2019 coronavirus disease in a non-Wuhan area of Hubei Province, China: a retrospective study. *BMC Infect Dis.* 2020;20(1):311.

104. Zhou Z, Zhao N, Shu Y, Han S, Chen B, Shu X. Effect of gastrointestinal symptoms on patients infected with COVID-19. *Gastroenterology.* 2020.

105. Zhou S, Wang Y, Zhu T, Xia L. CT Features of Coronavirus Disease 2019 (COVID-19) Pneumonia in 62 Patients in Wuhan, China. *AJR Am J Roentgenol.* 2020;214(6):1287-1294.

106. Zhou F, Yu T, Du R, et al. Clinical course and risk factors for mortality of adult inpatients with COVID-19 in Wuhan, China: a retrospective cohort study. *Lancet.* 2020;395(10229):1054-1062.

107. Zhu H, Wang L, Fang C, et al. Clinical analysis of 10 neonates born to mothers with 2019-nCoV pneumonia. *Transl Pediatr.* 2020;9(1):51-60.

108. Zhu Z, Cai T, Fan L, et al. Clinical value of immune-inflammatory parameters to assess the severity of coronavirus disease 2019. *International Journal of Infectious Diseases.* 2020;95:332-339.
